# Valuation of the EQ-5D-3L in Jordan

**DOI:** 10.1007/s10198-024-01712-z

**Published:** 2024-09-03

**Authors:** Abeer Al Rabayah, Bram Roudijk, Fredrick Dermawan Purba, Fanni Rencz, Saad Jaddoua, Uwe Siebert

**Affiliations:** 1 Institute of Public Health, Medical Decision Making and Health Technology Assessment, Department of Public Health, Health Services Research, and Health Technology Assessment, UMIT TIROL - University for Health Sciences and Technology, Hall in Tirol, Austria; 2 EuroQol Research Foundation, Rotterdam, The Netherlands; 3 Faculty of Psychology, Padjadjaran University, Bandung, Indonesia; 4 Department of Health Policy, Corvinus University of Budapest, Budapest, Hungary; 5 Pharmacy Department, King Hussein Cancer Center, Amman, Jordan; 6 Institute of Public Health, Medical Decision Making and Health Technology Assessment, Department of Public Health, Health Services Research, and Health Technology Assessment, UMIT TIROL-University for Health Sciences and Technology, Hall in Tirol, Austria; 7 Division of Health Technology Assessment, ONCOTYROL-Center for Personalized Cancer Medicine, Innsbruck, Austria; 8 Center for Health Decision Science, Departments of Epidemiology and Health Policy & Management, Harvard T.H. Chan School of Public Health, Boston, MA USA; 9 Institute for Technology Assessment and Department of Radiology, Massachusetts General Hospital, Harvard Medical School, Boston, MA USA; 10 Center for Drug Policy and Technology Assessment, Pharmacy Department, King Hussein Cancer Center, Amman, Jordan

**Keywords:** EQ-5D-3L, Jordan, Cost-utility, Virtual interviews, Valuation, Hybrid model, I19

## Abstract

**Background:**

In Jordan, no national value set is available for any preference-accompanied health utility measure.

**Objective:**

This study aims to develop a value set for EQ-5D-3L based on the preferences of the Jordanian general population.

**Methods:**

A representative sample of the Jordanian general population was obtained through quota sampling involving age, gender, and region. Participants aged above 18 years were interviewed via videoconferencing using the EuroQol Valuation Technology 2.1 protocol. Participants completed ten composite time trade-offs (cTTO) and ten discrete choice experiments (DCE) tasks. cTTO and DCE data were analyzed using linear and logistic regression models, respectively, and hybrid models were applied to the combined DCE and cTTO data.

**Results:**

A total of 301 participants with complete data were included in the analysis. The sample was representative of the general population regarding region, age, and gender. All model types applied, that is, random intercept model, random intercept Tobit, linear model with correction for heteroskedasticity, Tobit with correction for heteroskedasticity, and all hybrid models, were statistically significant. They showed logical consistency in terms of higher utility decrements with more severe levels. The hybrid model corrected for heteroskedasticity was selected to construct the Jordanian EQ-5D-3L value set as it showed the best fit and lowest mean absolute error. The predicted value for the most severe health state (33333) was − 0.563. Utility decrements due to mobility had the largest weight, followed by anxiety/depression, while usual activities had the smallest weight.

**Conclusion:**

This study provides the first EQ-5D-3L value set in the Middle East. The Jordanian EQ-5D-3L value set can now be used in health technology assessments for health policy planning by the Jordanian health sector’s decision-makers.

**Supplementary Information:**

The online version contains supplementary material available at 10.1007/s10198-024-01712-z.

## Introduction

Jordan is moving towards Universal Health Coverage (UHC) to fulfill the sustainable development goals by 2030 [1–3]. Therefore, there is an increased need to use health technology assessment (HTA) as a priority-setting tool to inform and guide policy decisions [4]. Using economic evaluation metrics as one component of HTA enables decision-makers to compare the value of different health technologies. Cost-utility analysis is considered the most suitable analysis type because it uses quality-adjusted life years (QALYs) as its standardized outcome metric [5]. QALYs reflect both quantity and quality of life. Estimating QALYs requires adjusting life years by health-related quality of life using a specific weight called utility value. Utilities represent health state values which indicate people's preferences. The utility scale is anchored on 0 (dead) and 1 (full health). Moreover, negative values are possible and represent health states worse than being dead [5]. In addition to economic evaluation, QALYs are an important outcome integrating benefits and harms expressed in mortality and health-related quality of life informing clinical guideline development [6]

Utilities can be generated indirectly using generic preference-accompanied measures [7]. Usually, the instruments consist of two components: a descriptive system and a value set that assigns a utility value to all possible health states of the descriptive system. Value sets are typically generated based on directly eliciting utilities using direct preference elicitation methods, such as time trade-off, standard gamble, or discrete choice experiment [8, 9]. The EQ-5D, which has two versions for adults (EQ-5D-3L and EQ-5D-5L), is the most used generic preference-accompanied measure [8]. It showed good psychometric properties in various health conditions and treatments [10]. Its descriptive system comprises five health dimensions: mobility, self-care, usual activities, pain/discomfort, and anxiety/depression. The EQ-5D-3L includes three levels of severity, while the EQ-5D-5L version includes five levels of severity for each dimension. Health profiles are described by answering each dimension of the descriptive system by selecting the severity level under each dimension that matches the respondent’s health status on that day. After answering all dimensions, a five-digit profile number that ranges from one to three or one to five, depending on the used EQ-5D version, may be generated. Profile numbers can be translated to a single index number (utility) using a value set generated from national valuation studies [11, 12]. Utilities might not be transferable from one country to another; previous studies have shown that countries with different religions, cultures, socioeconomic factors, and other characteristics can have different utilities based on their health state preferences [9, 13–15].

A previously published research based on the SNAPSHOT program in the Middle East region used utility values that were based on the UK value set due to the absence of country-specific utility value sets for EQ-5D in the Middle East countries [16]. The study highlighted a need for national value sets for these countries [16]. A systematic review of health state valuation in low middle-income countries showed that utility values are not available in most of these countries, and more health state valuation studies are needed to estimate locally relevant QALYs [17]. Two countries from the Middle East and North Africa (MENA) region, Tunisia and Iran, have generated their EQ-5D-3L value sets. However, their values reflect the Tunisian and Iranian population preferences, which might not be transferable to the Jordanian population [18, 19]. Previous studies showed that even in countries with comparable cultures and income levels, using non-country specific values significantly impacted incremental cost-effectiveness ratios (ICERs) and probabilistic sensitivity analysis results [20].

As the validity and reliability of the EQ-5D-3L Arabic Jordanian version have been assessed [21], our study aimed to generate a national EQ-5D-3L value set for Jordan based on the EuroQoL-Valuation Technology (EQ-VT) protocol [22]. Having a national value set is expected to generate utilities with country-specific validity, which can therefore be used in the economic evaluation of health technologies, clinical studies, population health surveys, and routine outcome measurements specific for Jordan [23].

## Methods

### Study design

This study was a national survey that consisted of two utility elicitation techniques: the composite time trade-off (cTTO) and the discrete choice experiment (DCE). Both techniques were based on the latest EQ-5D-5L EuroQol Group valuation protocol (EQ-VT v2.1) [24, 25]. The EQ-VT v2.1 has also been used for EQ-5D-3L valuation studies [18, 26]. Because of the COVID-19 pandemic, the study data collection phase was conducted online with an interviewer present via a videoconference platform. The Jordanian Arabic version of the EQ-VT was programmed using the Limesurvey, a web-based platform.

The standardized EQ-VT protocol consists of eight parts [25]: (i) welcome and study introduction, (ii) self-completed EQ-5D-3L and background questions, (iii) cTTO wheelchair example (introduction and two examples: states better than dead (BTD), and states worse than dead (WTD), (iv) three practice states (mild, severe, intermediate), (v) ten cTTO tasks, (vi) feedback module and structured feedback on the participant’s experience, (vii) ten DCE tasks where participants chose the best of two health states, (viii) structured feedback on the participants’ experience of DCE.

cTTO includes both the traditional TTO method for health states better than dead (BTD) and the lead-time TTO for health states worse than dead (WTD). Details on the cTTO are explained elsewhere [24]. The same health state design was used as in previous EQ-5D-3L valuation studies in Tunisia and Russia [18, 26]. For the cTTO task, 28 health states were selected for valuation and were distributed among three blocks. The worst health state (‘33333’) was included within each block, making the total number of ten health states per block. Moreover, each block included at least one mild state (four dimensions with no problems and only one with some problems, e.g., 11112) [24]. Each participant valued one block of health states using cTTO. The feedback module was included in the interview, which displays the implied ranking of the 10 states valued using cTTO from participants’ responses to the cTTO tasks. Participants could mark (‘flag’) the state(s) that they considered did not have the correct position [27].

For DCE health states, 60 pairs of health states were selected. They were generated using a bayesian efficient design approach and were distributed among six blocks (each block includes ten pairs of health states) [28]. The software EQ-VT was used to randomly assign participants to cTTO and DCE blocks of the health states [24, 25].

### Ethical approval

The study was reviewed and approved by the Institutional Review Board (IRB) at the King Hussein Cancer Center, Amman, Jordan (Approval number:21 KHCC 054). Also, the study was approved by the Research Committee for Scientific Ethical Questions (RCSEQ) at UMIT TIROL—University for Health Sciences and Technology, Hall in Tirol, Austria (Approval number 2986).

### Participants

The required sample size for the EQ-5D-3L valuation study is 300 participants [29, 30]. As the study follows a hybrid experimental design that combines cTTO and DCE tasks, the number of 300 respondents required to obtain significant statistical estimates was considered sufficient and similar to other EQ-5D-3L valuation studies (e.g., Russia and Tunisia) [18, 26].

The sampling method consisted of quota sampling based on region, gender, and age. The national population census from the Jordanian Department of Statistics (DOS) was used to achieve the required percentage for quota sampling. DOS is a governmental institution and authorized agency for releasing national census and statistical indicators for Jordan [31]. Jordan is divided administratively into twelve governorates, so we adopted the same twelve governorates to reach the representativeness of the population.

A global marketing research company was tasked to recruit the participants to achieve the required sample size of 300 according to the abovementioned quotas. To compensate for expected dropouts of up to 25%, a sample size of 400 was targeted. The marketing research company contacted potential participants and took their first verbal consent, and then provided the study team with a list of participants with their contact information. The study team then contacted the potential participant and scheduled an interview based on the participant’s preferred date and time. During this call, the knowledge of videoconferencing was assessed by interviewers. If any participant had a challenge with using videoconferencing, an educational video was sent to that participant along with providing education and instruction during the day of the interview. In the case of elderly participants, interviewers asked for family members who are familiar with videoconferencing to help in opening the virtual interview link. During the day of the virtual interview, participants consented for the second time, and we documented their agreement using the study software.

Participants were considered as drop out if they could not be reached after three call attempts at different times by the interviewers [32], refused to participate after providing initial consent to the marketing research company, and if participants did not show up on the day of the interview after confirmed scheduling. After completing the virtual interview, participants received an incentive of 20 Euros through a shopping voucher.

### Quality assurance and control

The interviews were conducted by a team of four interviewers. All are from health-related educational backgrounds, with a BSc degree in pharmacy and postgraduate degrees in pharmaceutic science, health economics, and health technology assessment. A standardized 1.5-day training workshop was provided by the EQ-VT Support Team at the EuroQol Office, and a 1.5-month pilot phase (practice interviews) before starting the real data collection [24, 25]. All interviews were checked (i.e., compliance with the study protocol, interviewers’ effect, and face validity) using a quality control tool developed by the EuroQol Group, and regular discussion sessions were held with members of the EQ-VT Support Team to maintain the quality of data being collected. In short, there were four Quality Control (QC) indicators [33]:

1. No explanation of the worse-than-dead task (lead-time TTO) for the wheelchair example.
2. Too short time spent on the wheelchair examples (less than 3 min).
3. Clear inconsistency in the cTTO ratings (33333 is not the lowest and at least 0.5 higher than the state with the lowest value)
4. Too short time for completing the cTTO task (total time for the 10 cTTO tasks less than 5 min)

Meeting any of the four QC indicators meant that the interview was of a suspected quality, and it was flagged in the QC report. Interviewers who produced four flagged interviews out of the first ten were re-trained by the principal investigator (PI) according to the EQ-5D valuation study code of conduct.

### Statistical analysis

#### Descriptive analysis

Descriptive statistics were computed for the respondents’ sociodemographic characteristics (gender, age, governorate of the respondent, and area type), and these were compared with the national census. Respondents' self-reported health was reported as a relative frequency of responses to each level of the EQ-5D-3L. EQ-VAS values were reported as mean and standard deviation (SD). The statistical analysis was conducted using STATA version 17.

#### Modeling the cTTO and DCE data

The cTTO and DCE data were modeled independently, as well as in combination, by using a hybrid model [34–36]. The hybrid model is a regression model that includes cTTO continuous and DCE binary data [34]. For all models, indicator variables for the level-dimension combinations were used as independent variables (mobility 2, mobility 3, self-care 2, self-care 3, usual activities 2, usual activities 3, pain/discomfort 2, pain/discomfort 3, anxiety/depression 2, anxiety/depression 3), with no problems (level 1) being used as the reference category. The estimated coefficients represent the utility decrement of moving from level 1(no problems) to level 2 (some problems) and from level 1 (no problems) to level 3 (most severe problems). Equation 1 shows the general regression equation form:

1 $$Y= \upbeta 0+\upbeta 1\times MO2+\upbeta 2\times MO3+\upbeta 3\times SC2+\upbeta 4\times SC3+\upbeta 5\times UA2+\upbeta 6\times UA3 +\upbeta 7\times PD2 +\upbeta 8\times PD3+\upbeta 9\times \text{AD}2+\upbeta 10\times AD3$$

With Y being the disutility (1-cTTO utility value for health state), ß being the regression coefficients, representing utility decrements MO2: mobility dimension level 2, MO3: mobility dimension level 3, SC2: self-care dimension level 2, SC3: self-care dimension level 3, UA2: usual activity dimension level 2, UA3: usual activity dimension level 3, PD2: pain/discomfort dimension level 2, PD3: pain/discomfort dimension level 3, AD2: Anxiety/depression dimension level 2, AD3: Anxiety/depression dimension level 3.

Linear regression models were used to model the cTTO data. As each respondent completed ten TTO tasks, a correlation between responses for each respondent is possible, and therefore, the cTTO data may be considered nested. Therefore, when modeling the cTTO data independently, a randomintercept model is used, allowing the intercept to vary across respondents. Furthermore, due to the way in which the cTTO task is constructed, respondents can only trade-off up to 20 years to avoid an impaired health state, leading to a truncated response scale with a minimum value of − 1. However, respondents may consider some health states to have a lower value than − 1, and may be willing to trade more years, had this been possible in the cTTO task. Therefore, the cTTO data are considered left censored, and a Tobit model can be used assuming a distribution beyond a threshold value (point of left censoring in our case, − 1).

As the responses to the cTTO tasks show more variation for more severe health states as compared to milder health states, the homoscedasticity assumption may be violated, which may bias the estimates. To address this issue of different errors according to health states' severity level, a model that accounts for heteroscedasticity was estimated on the cTTO data [34, 35].

The DCE data was analyzed using a logistic regression model. Modeling the DCE data produces values on a latent scale, which was rescaled using the theta parameter produced by the hybrid models to generate health state values on the QALY scale [28]. The theta parameter is calculated by hybrid models to standardize the coefficients coming from the logistic regression and linear regression. Therefore, a transformed logistic regression coefficient is generated by dividing the originally generated logistic regression coefficient by the theta parameter.

To combine the cTTO and DCE data into a single model, hybrid modeling was conducted, in which a joint likelihood function is estimated for the cTTO and DCE data [34–36]. Four different hybrid models were estimated: Standard Hybrid (assuming a likelihood function similar to that of linear regression for the cTTO data and a likelihood function similar to a logistic regression model for the DCE data), Hybrid Tobit model (assuming a Tobit link function for the cTTO data), Hybrid corrected for heteroscedasticity (HYBRID HET) (assuming a variance function of the error term for the cTTO data that is dependent on the ten independent variables of the main model), Hybrid Tobit corrected for heteroscedasticity (assuming a Tobit link function as well as a specific variance function for the error term of the cTTO data). All analyses were repeated after removing responses flagged by the respondents in the feedback module and removing non-traders as a sensitivity analysis.

#### Model performance and value set selection

Model performance was assessed regarding logical consistency and significance of parameter estimates, the goodness of fit using the Akaike Information Criterion AIC and BIC Bayesian Information Criterion measures, and prediction accuracy using the mean absolute error (MAE) and root mean square error (RMSE) [34, 35]. The model that performs best on these criteria was selected for the final value set.

#### Crosswalk EQ-5D-5L value set

In countries with no EQ-5D-5L value set yet, the mapping approach (cross-walk) can be used to generate an EQ-5D-5L value set from the EQ-5D-3L value set. The mapping approach is a function that predicts probabilities for a response to the EQ-5D-5L descriptive system, given their response to the EQ-5D-3L descriptive system. Using the full matrix of these conditional probabilities, it is possible to generate a value set for the EQ-5D-5L by multiplying the vector of EQ-5D-3L values with the probability matrix. In our study, we applied the mapping algorithm that has been developed by Van Hout et al. to generate an interim EQ-5D-5L value set for Jordan [37].

## Results

### Data collection and protocol compliance

A total of 301 respondents completed the valuation study interviews in Jordan from the 28th of September 2021 to the 2nd of April 2022. A total of 300/301 respondents passed all four QC criteria. The mean interview duration was 52 min. The mean time taken to complete a single TTO task was 132 s, and the meantime taken to complete a single DCE task was 40 s. The mean number of iterative steps (moves) needed to complete the cTTO real health states task was 11 moves. Overall, 95 (3.16%) health states out of 3010 were flagged. The proportion of interviews with overall inconsistencies was 9% before the feedback module, which decreased to 7% after the feedback module.

### Respondents’ characteristics

The study sample was representative of the general population in Jordan in terms of the governorate, gender, age, and area type, with a close estimate of the national census. Urban areas were overrepresented by 5% compared to the census data [38] (Table 1).

**Table 1:** Characteristics of respondents

Characteristic	Number (n)	Percentage (%)	General population (%)	Proportional difference (%)
Gender				
Male	154	51.16	*51.00	+ 0.16
Female	147	48.84	*49.00	– 0.16
Age				
18–24	54	17.94	*21.00	– 3.06
25–29	41	13.62	*11.00	+ 2.62
30–34	30	9.96	*10.00	– 0.04
35–39	28	9.30	*10.00	– 0.7
40–44	31	10.29	*10.00	+ 0.29
45–49	27	8.97	*9.00	– 0.03
50–54	28	9.30	8.00	+ 1.30
55–59	18	5.98	6.00	– 0.02
60–64	20	6.64	5.00	+ 1.64
65 +	24	7.97	10.00	– 2.03
Governorates				
Amman	121	40.20	*39.00	+ 1.2
Irbid	61	20.27	*20.00	+ 0.27
Zarqa	44	14.62	*14.00	+ 0.62
Balqa	18	5.98	*6.00	– 0.02
Al-Mafraq	13	4.32	*5.00	– 0.68
Jerash	9	2.99	*3.00	– 0.01
Kerak	8	2.66	*4.00	– 1.34
Madaba	6	1.99	*2.00	– 0.01
Ajloun	6	1.99	*2.00	– 0.01
Aqaba	6	1.99	*2.00	– 0.01
Ma'an	6	1.99	*2.00	– 0.01
Tafilah	3	1.00	*1.00	0
Area type				
Urban	264	93	*88.00	+ 5.00
Rural	36	7	*12.00	– 5.00
Education level				
Less than secondary	21	6.97	**22.00	– 15.03
Secondary	79	26.25	**40.10	– 13.85
Intermediate diploma	51	16.94	**9.50	+ 7.44
Bachelor’s degree and above	150	49.84	**28.70	+ 21.14
Marital status				
Single	78	25.91	***40.03	– 14.12
Married	207	68.77	***55.48	+ 13.29
Divorced	4	1.33	***1.14	+ 0.19
Widowed	12	3.99	***3.09	+ 0.90
Employment				
Employed Jordanian	154	51.16	***25.16	
Unemployed	105	34.88	***24.10	
Retired	42	13.95	NA	
Employment sector				
Public sector	53	34.42	***38.80	
Private sector	80	51.95	***60.40	
Non-profit organizations	10	6.49	–	
Self employed	11	7.14	–	
Health insurance status				
Have insurance	231	76.74	***69.00	
No insurance	70	23.26	***31.00	
Health insurance type				
Ministry of Health	99	42.86	***41.70	
Royal medical services	53	22.94	***38.00	
Universities’ Hospitals	10	4.33	***2.50	
Private insurance	46	19.91	***12.40	
UNRWA	1	0.43	***2.50	
Others	22	9.52	***2.90	

^*^Department of Statistics [38]

^**^Marketing research company (Jordanians above 18 years) [69],

***General Population and Housing Census 2015 [70], Note: The frequencies of the employment categories do not sum to 1

Overall, 69% were married, 50% of the respondents had high education, 51% were employed. Three-quarters (77%) of the respondents had health insurance (43% by the Ministry of Health (MOH) and 23% by RMS), and 28% had chronic diseases (Table 2). The mean (SD) EQ VAS value was 81.24 ± 13.90. Forty respondents had a health profile without any problems (11111). The highest proportion of health problems were reported in the pain/discomfort and anxiety/depression dimensions (Table 2).

**Table 2:** Distribution of EQ-5D-3L levels among respondents

EQ-5D dimension	Number (*n*)	Percentage (%)
Mobility		
Level 1: I have no problems in walking about	256	85.05
Level 2: I have some problems in walking about	45	14.95
Level 3 I am confined to bed	0	0
Self-care		
Level 1: I have no problems with self-care	297	98.67
Level 2: I have some problems washing or dressing myself	4	1.33
Level 3: I am unable to wash or dress myself	0	0
Usual activities		
Level 1: I have no problems with performing my usual activities	263	87.38
Level 2: I have some problems with performing my usual activities	35	11.63
Level 3: I am unable to perform my usual activities	3	1.00
Pain/discomfort		
Level 1: I have no pain or discomfort	180	59.80
Level 2: I have moderate pain or discomfort	115	38.21
Level 3: I have extreme pain or discomfort	6	1.99
Anxiety/depression		
Level 1: I am not anxious or depressed	195	64.78
Level 2: I am moderately anxious or depressed	98	32.56
Level 3: I am extremely anxious or depressed	8	2.66

### Descriptive statistics of observed cTTO values

**Fig. 1:**
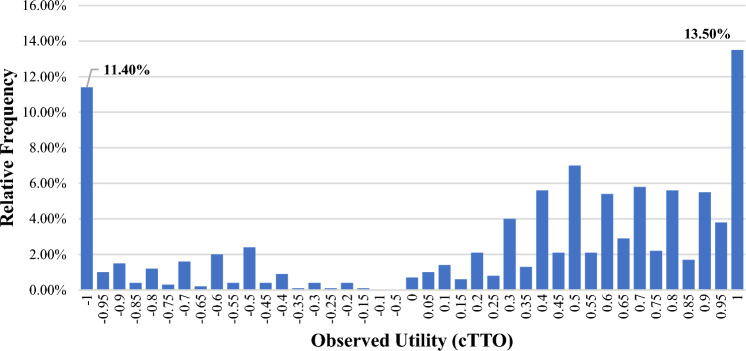
Distribution of the observed cTTO values in percentage. cTTO composite time-trade-off

The distribution of the observed cTTO values showed clustering at values − 1 (11%) and 1 (14%). No clustering was seen at zero, − 0.5, or 0.5 (Fig. 1). The mean observed value for the 28 health states was 0.290 ± 0.680, and the median was 0.5 with a minimum value of − 1 and a max value of 1. The pits state (33333) had the lowest observed cTTO mean value (− 0.541), and the highest mean value was 0.889 (11112). Six (2%) respondents were non-traders.

### Data modeling

The performance of all cTTO models showed logical consistency where utility decrements with level 3 in all dimensions being greater than the utility decrements with the less severe level 2 (Table 2). The parameter estimates in all four models were statistically significant (P value < 0.05). The constant (intercept) term was not statistically significant in all models; therefore, it was suppressed.

The AIC and BIC results of the cTTO models showed that the random intercept model produced the best data fit (lowest AIC and BIC estimates compared to the other three models). In addition, the random intercept model produced the lowest MAE and RMSE, indicating the most accurate predictions compared with the remaining three cTTO models (Table 3).

**Table 3:** Parameter estimates and fit statistics of the cTTO models

	Random intercept, MLE (Model 1) β (SE)	Tobit (Model 2) β (SE)	HET (Model 3) β (SE)	Tobit HET (Model 4) β (SE)
MO2	0.119 (0.020)	0.109 (0.023)	0.114 (0.017)	0.110 (0.016)
MO3	0.446 (0.020)	0.480 (0.022)	0.445 (0.025)	0.488 (0.030)
SC2	0.157 (0.018)	0.162 (0.020)	0.168 (0.018)	0.177 (0.018)
SC3	0.295(0.019)	0.321 (0.021)	0.303 (0.022)	0.342 (0.025)
UA2	0.098 (0.020)	0.097 (0.022)	0.085 (0.017)	0.084 (0.016)
UA3	0.185 (0.019)	0.203 (0.021)	0.183 (0.024)	0.193 (0.027)
PD2	0.094 (0.019)	0.091 (0.020)	0.084 (0.017)	0.079 (0.017)
PD3	0.315 (0.019)	0.337 (0.021)	0.313 (0.024)	0.338 (0.027)
AD2	0.115(0.020)	0.120 (0.022)	0.094 (0.017)	0.096 (0.017)
AD3	0.335 (0.019)	0.358 (0.021)	0.340 (0.024)	0.372 (0.027)
Uncensored Observations	3010	2671	3010	2671
Right-Censored Observations	0	339	0	339
AIC	3581.990	4424.607	4047.273	4539.967
BIC	3660.116	4502.733	4179.487	4672.180
MAE	0.423	0.431	0.423	0.715
RMSE	0.274	0.287	0.274	0.798
Predicted value for the pit state	– 0.569	– 0.683	– 0.587	– 0.731

Parameters are significant, P value < 0.0001

*cTTO* composite time trade-off, β coefficient, *SE* standard error, Dimensions: *MO2* mobility level 2, *MO3* mobility level 3, *SC2* self-care level 2, *SC3* self-care level 3, *UA2* usual activities level 2, *UA3* usual activities level3, PD2: pain/discomfort level 2, *PD3* pain/discomfort level 3, *AD2* anxiety/depression level 2, *AD3* anxiety/depression level 3., MLE maximum likelihood, HET model:interval liner regression correcting for heteroscedasticity, *AIC* akaike information criterion, BIC Bayesian Information Criterion, *MAE* Mean absolute error, *RMSE* Root mean square error, *MLE* maximum likelihood estimation

The parameter estimates for the DCE logistic model were rescaled using the theta parameter [34]. The generated coefficients were statistically significant and logically ordered. The weights were reported in the same ranking as the cTTO models, with the mobility dimension having the largest utility decrement (Table 4).

**Table 4:** Parameter estimates and fit statistics of the DCE models

EQ-5D-3L dimension	Conditional Logit for DCE data (Model 1)		Rescaled β
	β	SE	
MO2	0.505	0.079	0.122
MO3	2.134	0.102	0.515
SC2	0.743	0.075	0.179
SC3	1.191	0.089	0.288
UA2	0.396	0.079	0.096
UA3	0.434	0.082	0.105
PD2	0.332	0.072	0.080
PD3	1.140	0.088	0.275
AD2	0.459	0.083	0.111
AD3	1.403	0.094	0.339

*DCE* discrete choice experiment, β coefficient, *SE* standard error, Dimensions: *MO2* mobility level 2, *MO3* mobility level 3, *SC2* self-care level 2, *SC3* self-care level 3, *UA2* usual activities level 2, *UA3* usual activities level3, *PD2* pain/discomfort level 2, *PD3* pain/discomfort level 3, *AD2* anxiety/depression level 2, *AD3* anxiety/depression level 3

All parameters were significant, P value < 0.0001

The performance of all hybrid models showed logical consistency. The parameter estimates in all four models were statistically significant (P value < 0.0001). The constant term was not significant in all models; therefore, it was suppressed (Table 5).

**Table 5:** Parameter estimates and fit statistics of the hybrid models

EQ-5D Level	HYBRID (Model I) β (SE)	HYBRID TOBIT (Model II) β (SE)	HYBRID HET (Model III) β (SE)	HYBRID TOBIT HET (Model IV) β (SE)
MO2	0.127 (0.014)	0.128 (0.015)	0.119 (0.012)	0.118 (0.012)
MO3	0.502 (0.014)	0.535 (0.016)	0.503 (0.015)	0.545 (0.017)
SC2	0.170 (0.013)	0.177 (0.014)	0.174 (0.012)	0.184 (0.012)
SC3	0.288 (0.014)	0.307 (0.015)	0.290 (0.013)	0.314 (0.015)
UA2	0.093 (0.014)	0.098 (0.016)	0.090 (0.011)	0.091 (0.011)
UA3	0.133 (0.014)	0.142 (0.015)	0.135 (0.014)	0.143 (0.015)
PD2	0.088 (0.013)	0.087 (0.014)	0.085 (0.011)	0.082 (0.011)
PD3	0.294 (0.014)	0.311 (0.015)	0.295 (0.013)	0.316 (0.015)
AD2	0.103 (0.014)	0.104 (0.015)	0.101 (0.012)	0.102 (0.012)
AD3	0.338 (0.013)	0.357 (0.014)	0.340 (0.013)	0.366 (0.015)
Uncensored observations	3010	2671	3010	2671
Right-Censored observations	0	339	0	339
AIC	7594.924	8434.783	7070.216	7565.229
BIC	7674.862	8514.720	7216.768	7710.781
MAE	0.388	0.392	0.387	0.392
RMSE	0.266	0.268	0.266	0.270
Predicted value for the pit state	− 0.555	− 0.651	− 0.563	− 0.684

Parameters are significant P value < 0.0001

β coefficient, SE standard error, Dimensions: *MO2*:mobility level 2, *MO3* mobility level 3, *SC2* self-care level 2, *SC3* self-care level 3, *UA2* usual activities level 2, UA3: usual activities level3, PD2: pain/discomfort level 2, *PD3* pain/discomfort level 3, *AD2* anxiety/depression level 2, *AD3* anxiety/depression level 3., HET regression model correcting for heteroscedasticity, *AIC* Akaike Information Criterion, *BIC* Bayesian Information Criterion, MAE Mean absolute error, *RMSE* Root mean square error

cTTO and DCE are two different methods, which are supposed to measure the same construct. We tested the agreement between modelled predictions using a scatterplot, and the agreement was good. The ordering of the level dimension combinations was similar between the two methods, which suggested as well that the data could be pooled.

**Fig. 2:**
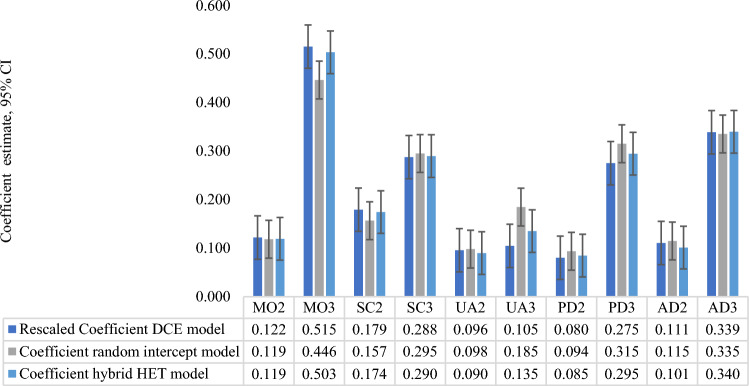
Utility decrement estimates (coefficient) from three best-fit models. *MO2* mobility level 2, *MO3* mobility level 3, *SC2* self-care level 2, *SC3* self-care level 3, *UA2* usual activities level 2, *UA3* usual activities level3, *PD2* pain/discomfort level 2, *PD3* pain/discomfort level 3, *AD2* anxiety/depression level 2, *AD3* anxiety/depression level 3. *DCE* discrete choice experiment, Hybrid HET regression model correcting for heteroscedasticity *CI* Confidence Interval

We present a comparison between the coefficient estimates of best-fit models in Fig. 2. It shows the best-fit cTTO model (random intercept model), the logistic regression model, and the best-fit hybrid model (HYBRID HET model).

The AIC and BIC results for the hybrid models showed that the HYBRID HET model produced the best data fit (lowest AIC and BIC estimates compared to the other three models). In addition, the HYBRID HET model produced the lowest MAE indicating the most accurate predictions compared with the remaining three hybrid models. However, the RMSE estimate was comparable to the HYBRID model (Table 6).

**Table 6:** Parameter estimates for the selected model: HYBRID HET

Parameters	Coefficient β(SE)		z	P > z	[95% Confidence interval]
MO2	0.119 (0.012)		9.99	0.000	0.096–0.143
MO3	0.503 (0.015)		34.68	0.000	0.475–0.532
SC2	0.174 (0.012)		14.86	0.000	0.151–0.197
SC3	0.290 (0.013)		21.56	0.000	0.263–0.316
UA2	0.090 (0.011)		7.83	0.000	0.067–0.112
UA3	0.135 (0.014)		9.92	0.000	0.108–0.162
PD2	0.085 (0.011)		7.49	0.000	0.063–0.107
PD3	0.295 (0.013)		21.99	0.000	0.268–0.320
AD2	0.101(0.012)		8.45	0.000	0.078–0.125
AD3	0.340 (0.013)		25.72	0.000	0.314–0.366

Parameters are significant P value < 0.0001

β coefficient, SE standard error, Dimensions: *MO2* mobility level 2, *MO3* mobility level 3, *SC2* self-care level 2, *SC3* self-care level 3, *UA2* usual activities level 2, *UA3* usual activities level3, *PD2* pain/discomfort level 2, PD3: pain/discomfort level 3, *AD2* anxiety/depression level 2, AD3: anxiety/depression level 3., *HET* hybrid regression model correcting for heteroscedasticity

The HYBRID HET jointly models the DCE and cTTO data, and corrects for heteroskedasticity in the cTTO data. The model predicted a value of − 0.563 for the pit state (33333), which is comparable to the mean observed cTTO value for this state (− 0.541). Furthermore, as the HYBRID HET model showed the best MAE over all models, both cTTO-only as well as the hybrid, the HYBRID HET model (Table 5) was selected as the Jordanian EQ-5D-3L value set (Eq. 2).

The predicted values for the 243 health states using Eq. 3 are presented in the online Supplementary Material (1).

2 $$U=1-0.119\times MO2-0.503\times MO3-0.174\times SC2-0.290\times SC3-0.090\times UA2-0.135\times UA3- 0.085\times PD2- 0.295\times PD3-0.101\times \text{AD}2-0.34\times \text{AD}3$$

In addition, the crosswalk EQ-5D-5L Jordanian values were generated using mapping methodology from the generated EQ-5D-3L value set [37]. The full value set is presented in Supplementary Material (2).

#### Sensitivity analysis results

The sensitivity analysis results showed that removing flagged responses in the feedback module did not change the data modelling results of the cTTO models. The random intercept model was the best fit for cTTO data (lowest AIC and BIC) and had the most accurate predictions (lowest MAE and RMSE) with and without flagged states. Also, the HYBRID HET remained the best-fitting model with the most precise predictions. Moreover, the modelling results were not impacted by removing the data of the six non-traders. Both the random intercept model and the HYBRID HET remained the best-fitting models.

## Discussion

Our study generated a Jordanian EQ-5D-3L value set based on a representative national sample following the EQ-VT protocol [24, 25]. We used two preference elicitation techniques: cTTO and DCE [24, 25]. Data were modeled using linear and logistic regressions based on the data type. In addition, hybrid models were employed to combine continuous cTTO data and dichotomous DCE data. Adjustments for censored data and heteroscedasticity were made during modeling. All indicator variables were statistically significant in all models. The hybrid model adjusted for heteroscedasticity was selected as the value set, given its superior performance in terms of goodness-of-fit and precision of predictions, including a closer prediction to the observed mean utility value of the pits state. Furthermore, the study allowed the generation of a crosswalk EQ-5D-5L value set based on previously developed methods [37].

When comparing the statistical effects of the different domain variables, our results showed that severe mobility problems had the highest impact on health-related quality of life based on Jordanian preferences, with a utility decrement of − 0.503 for being confined to bed. This result was consistent with previously published EQ-5D-3L valuation studies[18, 39–45] despite differences between valuation studies in terms of valuation and modeling methodology, time of data collection, and context. However, utility decrements per each EQ-5D dimension differed among countries in terms of magnitude. The utility decrement for being confined to bed was − 0.314 in the UK, − 0.430 in Spain, − 0.418 in Japan, − 0.411 in Denmark, − 0.558 in the USA, − 0.648 in Hungary, − 0.394 in Romania, and − 0.597 in Tunisia [18, 39–45]. These findings show that mobility, as a reflection of physical activity, is highly rated by people in many developed and developing countries. Unlike Jordan and Tunisia, the result of the Iranian value set showed that severe problems in self-care had the highest impact on health-related quality of life (− 0.235). However, Iran had a different valuation protocol and the study population covered only the capital of Iran [46]. As in many previously published valuation studies [18, 45–47], our study showed that severe problems in usual activities had the smallest impact on health-related quality of life with a utility decrement of (− 0.135).

Up to 2023, thirty-nine countries have conducted EQ-5D-3L valuation studies. However, only 8% of these studies were conducted in the Middle East and North Africa (MENA) [48]. Tunisia has an EQ-5D-3L value set, Egypt has an EQ-5D-5L value set, and  Saudi Arabia has recently published its EQ-5D-5L value sets [18, 49, 50]. Iran had two value sets; the EQ-5D-3L study did not rely on the EQ-VT protocol and its sample was not nationally representative [18, 19, 49, 51]. The number of valuation studies in the MENA region is expected to increase within the coming years. Value sets for Morocco, United Arab Emirates (UAE), and Lebanon are expected to be available within the coming future [52, 53]. This increased interest in valuation studies is moving parallel with the increased interest in HTA institutionalization in these countries [54].

Tunisia and Russia published their national EQ-5D-3L value sets in 2021; both studies applied the same study design and valuation protocol as our study [18, 26]. Therefore, our study is more comparable to these two studies than to other valuation studies. Table 7 compares these three value sets and how the predicted utility values for a selected health state differ across the three countries, reflecting different population preferences [18, 26]. As in Jordan, being confined to bed had the highest weight and impact on health-related quality of life in Tunisia and Russia. However, there were differences in the ranking of utility decrements of the remaining health dimensions. Severe anxiety/depression (-0.340) followed mobility in Jordan, while it was associated with the smallest disutility in Russia. In Tunisia, extreme anxiety/depression (-0.332) ranked third after being unable to wash or dress oneself [18, 26]. The high impact of anxiety/depression in Jordan might be explained within the context of the COVID-19 pandemic. According to the World Health Organization, the prevalence of anxiety and depression has increased by 25% since the start of the pandemic [55]. Despite the improvement in the mental health situation in 2021, there are still challenges in access to mental health support. Only 2% of health budgets are spent on mental health worldwide, with an even less percentage in low- and middle-income countries [55]. Therefore, health technologies that improve depression and anxiety might be a priority for Jordanians.

**Table 7:** Comparison between Tunisia, Jordan and Russia value sets

	Jordan (UMIC, MENA) HYBRID HET (Model III) β (SE)	Tunisia (LMIC, MENA) HYBRID HET (Model III) β (SE)	Russia (UMIC, Europe) HYBRID HET (Model III) β (SE)
MO2	0.119 (0.011)	0.076 (0.012)	0.041 (0.009)
MO3	0.503 (0.015)	0.597 (0.016)	0.458 (0.014)
SC2	0.174 (0.012)	0.165 (0.012)	0.075 (0.009)
SC3	0.290 (0.013)	0.340 (0.015)	0.246 (0.013)
UA2	0.090 (0.011)	0.078 (0.012)	0.073 (0.009)
UA3	0.135 (0.014)	0.251 (0.014)	0.242 (0.012)
PD2	0.085 (0.011)	0.057 (0.012)	0.066 (0.009)
PD3	0.295 (0.013)	0.276 (0.014)	0.377 (0.012)
AD2	0.101 (0.012)	0.095 (0.012)	0.041 (0.010)
AD3	0.340 (0.013)	0.332 (0.014)	0.179 (0.011)
Ordering of dimensions	MO-AD-PD-SC-UA	MO-SC-AD-PD-UA	MO-PD-SC-UA-AD
Predicted value for the pit state	− 0.563	**− **0.796	− 0.503
Health States 11223	0.485	0.533	0.682
12222	0.550	0.605	0.745
21321	0.661	0.356	0.535
33232	− 0.279	− 0.386	− 0.195
Month and year of data collection	September 2021–April 2022	June–September 2019	August–November 2019
Mode of administration	Virtual	Face to Face	Face to Face

β coefficient, *SE* standard error, Dimensions: *MO2* mobility level 2, *MO3* mobility level 3, *SC2* self-care level 2, *SC3* self-care level 3, *UA2* usual activities level 2, *UA3* usual activities level3, *PD2* pain/discomfort level 2, *PD3* pain/discomfort level 3, *AD2* anxiety/depression level 2, *AD3* anxiety/depression level 3., Hybrid HET regression model correcting for heteroscedasticity. *LMIC* lower middle-income country. *MENA* Middle East and North Africa, *UMIC* upper middle-income country

Our study differed in terms of mode of administration from the Tunisian and Russian studies. Face-to-face interview was the applied mode of administration in Tunisia and Russia, while in our study, we followed a virtual mode of administration using videoconferencing due to the COVID-19 pandemic. Our study is the first EQ-5D-3L valuation study that was conducted virtually. In Italy, the EQ-5D-5L valuation study showed that videoconferencing is a feasible mode of administration for EQ-5D valuation studies [56]. Our study provided insight into the feasibility of videoconferencing for valuation studies in low- and middle-income countries. It showed that using videoconferencing generated good quality data and can be a useful mode of administration option in upper-middle-income countries with access to the internet and the availability of computers [57, 58]. In Jordan, according to the national telecom and information technology prevalence and usage survey in homes, 27% of households have a computer (laptop or desktop), and the internet penetration rate is 86% [59]. The main reason that was stated for not having a computer is using smartphones [59]. Our study showed that 40% of households have computers. Two recent studies showed no difference in cTTO values across modes of administration, and both generated good-quality data. Therefore, videoconferencing is considered feasible and acceptable [57, 58]. However, the preferences for each mode of administration differed according to respondents’ characteristics [57, 58].

A total of 11% of the observed cTTO values was -1 reflecting the lowest possible value according to the EQ-VT protocol. Our results were comparable to Tunisia (12%) but different from Russia (7%) and Hungary (4%) [18, 26, 60]. Upon looking at the EQ-5D-5L for Egypt and Ethiopia, the clustering at -1 was 13% and 8%, respectively [49, 61]. Jordan, Tunisia, Egypt, and Ethiopia are classified as low- and middle-income countries [62]. There was a higher percentage of -1 values in those low- and middle-income countries which might reflect that the population in those countries is willing to trade off all their life years to avoid living in some conditions that they perceive as extremely worse than dead. This finding might be explained within the context of how the population perceives provided health services, including caregiving, in their countries. In addition, in Jordan, families are considered the primary caregivers, and people might feel that being sick might burden their families.

Our study has several strengths. First, the virtual mode of interview administration facilitated the study logistics. The interview was conducted while participants were fully focused during the interview without distractions that we might have encountered if the interview had been conducted in a public place. The data quality was high, with only one reported flagged interview. However, one limitation of using virtual interviews was the availability of computers, as many people prefer to use just smartphones rather than computers. In addition, the penetration rate of the internet needs to be checked in countries before deciding to apply for virtual study. On the other hand, we achieved most of the elderly quota despite the virtual nature of our study. In Jordanian culture, usually, the young generation helps the older generation in videoconferencing. In addition, during the COVID-19 pandemic, Jordanians have become more familiar with using videoconferencing for homeschooling, and this helped us in introducing the virtual nature of our study. Second, our study achieved excellent representativeness, making our results generalizable. Third, we applied the most recent EuroQol valuation protocol, including all modules (cTTO, DCE, and feedback module), which makes our study standardized and comparable to other countries.

On the other hand, our study encountered several limitations. First, as our study was conducted during the COVID-19 pandemic, we do not know whether and how the pandemic impacted the results. However, this is a challenge with all valuation studies that collected data in this time. This challenge might be an area that requires further investigation [63]. Second, despite the fact that the virtual nature of our study led to some strengths, it was also associated with some limitations. Excluding respondents who do not have computers might have impacted the composition of our study population. However, our sample was representative or closely representative of the general population regarding age, gender, governorate, and urban/rural residence. Nevertheless, respondents with higher education (bachelor’s degree and above) were high in our study (50%). This high percentage might be explained within the context of the virtual nature of our study, where the probability of having a computer is higher among more educated people [38]. On the other hand, some previously published valuation studies conducted face-to-face also had a higher percentage of highly educated people, 57% in Tunisia and 42% in Iran [18, 46]. This phenomenon might be understood considering the nature of valuation studies that require cognitive abilities and high engagement. Furthermore, there was a slight over-representation of married respondents and an underrepresentation of single respondents in our study. This situation might reflect the differences in the willingness to participate in surveys based on the sociodemographic characteristics of potential participants. For example, in Norway never married people had a lower probability to participate in surveys compared to married people [64]. However, the evidence regarding education and marital status, is mixed, where some studies found these variables affecting utilities, while others did not [65–68]

## Recommendations, policy implications, future direction

Previous studies showed that using different value sets with different magnitudes of utility decrement impacts the calculations of QALYs with reflection on incremental cost effectiveness ratios (ICERs). Table 7 shows an example of how one health state was valued differently according to the used value set. The chosen health state “11223” had the lowest value in Jordan, followed by Tunisia and Russia. This comparison shows how expected QALYs for courses of diseases with and without interventions will differ between those three countries. However, investigating how those changes impact policy decisions, and final reimbursement mix is essential.

## Conclusion

This valuation study generated the first EQ-5D-3L value set in Jordan based on a nationally representative general population sample. It provides decision makers with the required scoring algorithm to generate locally relevant QALYs based on the Jordanian population preferences and support HTA implementation in Jordan, and other countries in the MENA region without national value sets.

## Supplementary Information

Below is the link to the electronic supplementary material.

Supplementary file1 (DOCX 104 KB)

## Data Availability

Data from the corresponding author upon a reasonable request.
