# Supplementary material for: Valuation of the EQ-5D-3L in Jordan
Source: Eur J Health Econ. 2024 Sep 3;26(3):487–501. doi: 10.1007/s10198-024-01712-z (PMC11937146; doi:10.1007/s10198-024-01712-z)
Supplement: Supplementary file 1 — Supplementary file1 (DOCX 104 KB) [file 10198_2024_1712_MOESM1_ESM.docx]

**Supplementary material (1)**

Supplementary material (1): Jordanian value set for the EQ-5D-3L

| Health state | | Utility value | Health state | Utility value | Health state | Utility value |  |
| --- | --- | --- | --- | --- | --- | --- | --- |
| 11111 | | 1.000 | **21111** | 0.881 | **31111** | 0.497 |  |
| 11112 | | 0.899 | **21112** | 0.780 | **31112** | 0.396 |  |
| 11113 | | 0.660 | **21113** | 0.541 | **31113** | 0.157 |  |
| 11121 | | 0.915 | **21121** | 0.796 | **31121** | 0.412 |  |
| 11122 | | 0.814 | **21122** | 0.695 | **31122** | 0.311 |  |
| 11123 | | 0.575 | **21123** | 0.456 | **31123** | 0.072 |  |
| 11131 | | 0.705 | **21131** | 0.586 | **31131** | 0.101 |  |
| 11132 | | 0.604 | **21132** | 0.485 | **31132** | 0.101 |  |
| 11133 | | 0.365 | **21133** | 0.246 | **31133** | -0.138 |  |
| 11211 | | 0.910 | **21211** | 0.791 | **31211** | 0.407 |  |
| 11212 | | 0.809 | **21212** | 0.690 | **31212** | 0.306 |  |
| 11213 | | 0.570 | **21213** | 0.451 | **31213** | 0.067 |  |
| 11221 | | 0.825 | **21221** | 0.706 | **31221** | 0.322 |  |
| 11222 | | 0.724 | **21222** | 0.605 | **31222** | 0.221 |  |
| 11223 | | 0.485 | **21223** | 0.366 | **31223** | -0.018 |  |
| 11231 | | 0.615 | **21231** | 0.496 | **31231** | 0.112 |  |
| 11232 | | 0.514 | **21232** | 0.395 | **31232** | 0.011 |  |
| 11233 | | 0.275 | **21233** | 0.156 | **31233** | -0.228 |  |
| 11311 | | 0.865 | **21311** | 0.746 | **31311** | 0.362 |  |
| 11312 | | 0.764 | **21312** | 0.645 | **31312** | 0.261 |  |
| 11313 | | 0.525 | **21313** | 0.406 | **31313** | 0.022 |  |
| 11321 | | 0.780 | **21321** | 0.661 | **31321** | 0.277 |  |
| 11322 | | 0.679 | **21322** | 0.560 | **31322** | 0.176 |  |
| 11323 | | 0.440 | **21323** | 0.321 | **31323** | -0.063 |  |
| 11331 | | 0.570 | **21331** | 0.451 | **31331** | 0.067 |  |
| 11332 | | 0.469 | **21332** | 0.350 | **31332** | -0.034 |  |
| 11333 | | 0.230 | **21333** | 0.111 | **31333** | -0.273 |  |
| 12111 | | 0.826 | **22111** | 0.707 | **32111** | 0.323 |  |
| 12112 | | 0.725 | **22112** | 0.606 | **32112** | 0.222 |  |
| 12113 | | 0.486 | **22113** | 0.367 | **32113** | -0.017 |  |
| 12121 | | 0.741 | **22121** | 0.521 | **32121** | 0.238 |  |
| Health state | | | **Utility value** | **Health state** | **Utility value** | **Health state** | **Utility value** |
| 12122 | | 0.640 | **22122** | 0.521 | **32122** | 0.137 |  |
| 12123 | | 0.401 | **22123** | 0.282 | **32123** | -0.102 |  |
| 12131 | | 0.531 | **22131** | 0.412 | **32131** | 0.028 |  |
| 12132 | | 0.430 | **22132** | 0.311 | **32132** | -0.073 |  |
| 12133 | | 0.191 | **22133** | 0.072 | **32133** | -0.312 |  |
| 12211 | | 0.736 | **22211** | 0.617 | **32211** | 0.233 |  |
| 12212 | | 0.635 | **22212** | 0.516 | **32212** | 0.132 |  |
| 12213 | | 0.396 | **22213** | 0.277 | **32213** | -0.107 |  |
| 12221 | | 0.651 | **22221** | 0.532 | **32221** | 0.148 |  |
| 12222 | | 0.550 | **22222** | 0.431 | **32222** | 0.047 |  |
| 12223 | | 0.311 | **22223** | 0.192 | **32223** | -0.192 |  |
| 12231 | | 0.441 | **22231** | 0.322 | **32231** | -0.062 |  |
| 12232 | | 0.340 | **22232** | 0.221 | **32232** | -0.163 |  |
| 12233 | | 0.101 | **22233** | -0.018 | **32233** | -0.402 |  |
| 12311 | | 0.691 | **22311** | 0.572 | **32311** | 0.188 |  |
| 12312 | | 0.590 | **22312** | 0.471 | **32312** | 0.087 |  |
| 12313 | | 0.351 | **22313** | 0.232 | **32313** | -0.152 |  |
| 12321 | | 0.606 | **22321** | 0.487 | **32321** | 0.103 |  |
| 12322 | | 0.505 | **22322** | 0.386 | **32322** | 0.002 |  |
| 12323 | | 0.266 | **22323** | 0.147 | **32323** | -0.237 |  |
| 12331 | | 0.396 | **22331** | 0.277 | **32331** | -0.107 |  |
| 12332 | | 0.295 | **22332** | 0.176 | **32332** | -0.208 |  |
| 12333 | | 0.056 | **22333** | -0.063 | **32333** | -0.447 |  |
| 13111 | | 0.710 | **23111** | 0.591 | **33111** | 0.207 |  |
| 13112 | | 0.609 | **23112** | 0.490 | **33112** | 0.106 |  |
| 13113 | | 0.370 | **23113** | 0.251 | **33113** | -0.133 |  |
| 13121 | | 0.625 | **23121** | 0.506 | **33121** | 0.122 |  |
| 13122 | | 0.524 | **23122** | 0.405 | **33122** | 0.021 |  |
| 13123 | | 0.285 | **23123** | 0.166 | **33123** | -0.218 |  |
| 13131 | | 0.415 | **23131** | 0.296 | **33131** | -0.088 |  |
| 13132 | | 0.314 | **23132** | 0.195 | **33132** | -0.189 |  |
| 13133 | | 0.075 | **23133** | -0.044 | **33133** | -0.428 |  |
| 13211 | | 0.620 | **23211** | 0.501 | **33211** | 0.117 |  |
| 13212 | | 0.519 | **23212** | 0.400 | **33212** | 0.016 |  |
| Health state | | **Utility value** | | **Health state** | **Utility value** | **Health state** | **Utility value** |
| 13213 | | 0.280 | **23213** | 0.161 | **33213** | -0.223 |  |
| 13221 | | 0.535 | **23221** | 0.416 | **33221** | 0.032 |  |
| 13222 | | 0.434 | **23222** | 0.315 | **33222** | -0.069 |  |
| 13223 | | 0.195 | **23223** | 0.076 | **33223** | -0.308 |  |
| 13231 | | 0.325 | **23231** | 0.206 | **33231** | -0.178 |  |
| 13232 | | 0.224 | **23232** | 0.105 | **33232** | -0.279 |  |
| 13233 | | -0.015 | **23233** | -0.134 | **33233** | -0.518 |  |
| 13311 | | 0.575 | **23311** | 0.456 | **33311** | 0.072 |  |
| 13312 | | 0.474 | **23312** | 0.355 | **33312** | -0.029 |  |
| 13313 | | 0.235 | **23313** | 0.116 | **33313** | -0.268 |  |
| 13321 | | 0.490 | **23321** | 0.371 | **33321** | -0.013 |  |
| 13322 | | 0.389 | **23322** | 0.270 | **33322** | -0.114 |  |
| 13323 | | 0.150 | **23323** | 0.031 | **33323** | -0.353 |  |
| 13331 | | 0.280 | **23331** | 0.161 | **33331** | -0.223 |  |
| 13332 | | 0.179 | **23332** | 0.060 | **33332** | -0.324 |  |
| 13333 | | -0.060 | **23333** | -0.179 | **33333** | -0.563 |  |

**Supplementary material (2)**

Supplementary material (2) Jordanian cross walk value set for the EQ-5D-5L

| Health  state | Utility value | Health  state | Utility value | Health state | Utility value | Health  state | Utility value |
| --- | --- | --- | --- | --- | --- | --- | --- |
| 11111 | 1.000 | 11421 | 0.826 | 12231 | 0.695 | 12541 | 0.543 |
| 11112 | 0.920 | 11422 | 0.746 | 12232 | 0.615 | 12542 | 0.463 |
| 11113 | 0.899 | 11423 | 0.726 | 12233 | 0.594 | 12543 | 0.442 |
| 11114 | 0.783 | 11424 | 0.610 | 12234 | 0.478 | 12544 | 0.327 |
| 11115 | 0.663 | 11425 | 0.489 | 12235 | 0.358 | 12545 | 0.206 |
| 11121 | 0.931 | 11431 | 0.809 | 12241 | 0.612 | 12551 | 0.419 |
| 11122 | 0.851 | 11432 | 0.729 | 12242 | 0.532 | 12552 | 0.339 |
| 11123 | 0.830 | 11433 | 0.709 | 12243 | 0.512 | 12553 | 0.319 |
| 11124 | 0.715 | 11434 | 0.593 | 12244 | 0.396 | 12554 | 0.203 |
| 11125 | 0.594 | 11435 | 0.472 | 12245 | 0.275 | 12555 | 0.082 |
| 11131 | 0.914 | 11441 | 0.727 | 12251 | 0.488 | 13111 | 0.821 |
| 11132 | 0.834 | 11442 | 0.647 | 12252 | 0.408 | 13112 | 0.741 |
| 11133 | 0.813 | 11443 | 0.626 | 12253 | 0.388 | 13113 | 0.721 |
| 11134 | 0.697 | 11444 | 0.510 | 12254 | 0.272 | 13114 | 0.605 |
| 11135 | 0.577 | 11445 | 0.390 | 12255 | 0.151 | 13115 | 0.484 |
| 11141 | 0.832 | 11451 | 0.603 | 12311 | 0.763 | 13121 | 0.752 |
| 11142 | 0.752 | 11452 | 0.523 | 12312 | 0.683 | 13122 | 0.672 |
| 11143 | 0.731 | 11453 | 0.502 | 12313 | 0.663 | 13123 | 0.652 |
| 11144 | 0.615 | 11454 | 0.386 | 12314 | 0.547 | 13124 | 0.536 |
| 11145 | 0.495 | 11455 | 0.266 | 12315 | 0.426 | 13125 | 0.415 |
| 11151 | 0.708 | 11511 | 0.860 | 12321 | 0.695 | 13131 | 0.735 |
| 11152 | 0.628 | 11512 | 0.781 | 12322 | 0.615 | 13132 | 0.655 |
| 11153 | 0.607 | 11513 | 0.760 | 12323 | 0.594 | 13133 | 0.635 |
| 11154 | 0.491 | 11514 | 0.644 | 12324 | 0.478 | 13134 | 0.519 |
| 11155 | 0.371 | 11515 | 0.523 | 12325 | 0.358 | 13135 | 0.398 |
| 11211 | 0.930 | 11521 | 0.792 | 12331 | 0.677 | 13141 | 0.653 |
| 11212 | 0.850 | 11522 | 0.712 | 12332 | 0.598 | 13142 | 0.573 |
| 11213 | 0.829 | 11523 | 0.691 | 12333 | 0.577 | 13143 | 0.552 |
| 11214 | 0.713 | 11524 | 0.575 | 12334 | 0.461 | 13144 | 0.436 |
| 11215 | 0.593 | 11525 | 0.455 | 12335 | 0.340 | 13145 | 0.316 |
| 11221 | 0.861 | 11531 | 0.775 | 12341 | 0.595 | 13151 | 0.529 |
| 11222 | 0.781 | 11532 | 0.695 | 12342 | 0.515 | 13152 | 0.449 |
| 11223 | 0.760 | 11533 | 0.674 | 12343 | 0.494 | 13153 | 0.428 |
| 11224 | 0.644 | 11534 | 0.558 | 12344 | 0.378 | 13154 | 0.312 |
| 11225 | 0.524 | 11535 | 0.438 | 12345 | 0.258 | 13155 | 0.192 |
| 11231 | 0.844 | 11541 | 0.692 | 12351 | 0.471 | 13211 | 0.751 |
| 11232 | 0.764 | 11542 | 0.612 | 12352 | 0.391 | 13212 | 0.671 |
| 11233 | 0.743 | 11543 | 0.591 | 12353 | 0.370 | 13213 | 0.650 |
| 11234 | 0.627 | 11544 | 0.475 | 12354 | 0.254 | 13214 | 0.534 |
| 11235 | 0.507 | 11545 | 0.355 | 12355 | 0.134 | 13215 | 0.414 |
| 11241 | 0.761 | 11551 | 0.568 | 12411 | 0.746 | 13221 | 0.682 |
| 11242 | 0.681 | 11552 | 0.488 | 12412 | 0.666 | 13222 | 0.602 |
| 11243 | 0.661 | 11553 | 0.467 | 12413 | 0.646 | 13223 | 0.581 |
| 11244 | 0.545 | 11554 | 0.351 | 12414 | 0.530 | 13224 | 0.465 |
| 11245 | 0.424 | 11555 | 0.231 | 12415 | 0.409 | 13225 | 0.345 |
| 11251 | 0.637 | 12111 | 0.851 | 12421 | 0.677 | 13231 | 0.665 |
| 11252 | 0.557 | 12112 | 0.771 | 12422 | 0.598 | 13232 | 0.585 |
| 11253 | 0.537 | 12113 | 0.750 | 12423 | 0.577 | 13233 | 0.564 |
| 11254 | 0.421 | 12114 | 0.634 | 12424 | 0.461 | 13234 | 0.448 |
| 11255 | 0.300 | 12115 | 0.514 | 12425 | 0.340 | 13235 | 0.328 |
| 11311 | 0.912 | 12121 | 0.782 | 12431 | 0.660 | 13241 | 0.582 |
| 11312 | 0.832 | 12122 | 0.702 | 12432 | 0.581 | 13242 | 0.503 |
| 11313 | 0.812 | 12123 | 0.682 | 12433 | 0.560 | 13243 | 0.482 |
| 11314 | 0.696 | 12124 | 0.566 | 12434 | 0.444 | 13244 | 0.366 |
| 11315 | 0.575 | 12125 | 0.445 | 12435 | 0.323 | 13245 | 0.245 |
| 11321 | 0.843 | 12131 | 0.765 | 12441 | 0.578 | 13251 | 0.458 |
| 11322 | 0.763 | 12132 | 0.685 | 12442 | 0.498 | 13252 | 0.379 |
| 11323 | 0.743 | 12133 | 0.665 | 12443 | 0.477 | 13253 | 0.358 |
| 11324 | 0.627 | 12134 | 0.549 | 12444 | 0.361 | 13254 | 0.242 |
| 11325 | 0.506 | 12135 | 0.428 | 12445 | 0.241 | 13255 | 0.121 |
| 11331 | 0.826 | 12141 | 0.683 | 12451 | 0.454 | 13311 | 0.733 |
| 11332 | 0.746 | 12142 | 0.603 | 12452 | 0.374 | 13312 | 0.654 |
| 11333 | 0.726 | 12143 | 0.582 | 12453 | 0.353 | 13313 | 0.633 |
| 11334 | 0.610 | 12144 | 0.466 | 12454 | 0.237 | 13314 | 0.517 |
| 11335 | 0.489 | 12145 | 0.346 | 12455 | 0.117 | 13315 | 0.396 |
| 11341 | 0.744 | 12151 | 0.559 | 12511 | 0.712 | 13321 | 0.665 |
| 11342 | 0.664 | 12152 | 0.479 | 12512 | 0.632 | 13322 | 0.585 |
| 11343 | 0.643 | 12153 | 0.458 | 12513 | 0.611 | 13323 | 0.564 |
| 11344 | 0.527 | 12154 | 0.342 | 12514 | 0.495 | 13324 | 0.448 |
| 11345 | 0.407 | 12155 | 0.222 | 12515 | 0.375 | 13325 | 0.328 |
| 11351 | 0.620 | 12211 | 0.781 | 12521 | 0.643 | 13331 | 0.648 |
| 11352 | 0.540 | 12212 | 0.701 | 12522 | 0.563 | 13332 | 0.568 |
| 11353 | 0.519 | 12213 | 0.680 | 12523 | 0.542 | 13333 | 0.547 |
| 11354 | 0.403 | 12214 | 0.564 | 12524 | 0.426 | 13334 | 0.431 |
| 11355 | 0.283 | 12215 | 0.444 | 12525 | 0.306 | 13335 | 0.311 |
| 11411 | 0.895 | 12221 | 0.712 | 12531 | 0.626 | 13341 | 0.565 |
| 11412 | 0.815 | 12222 | 0.632 | 12532 | 0.546 | 13342 | 0.485 |
| 11413 | 0.794 | 12223 | 0.611 | 12533 | 0.525 | 13343 | 0.464 |
| 11414 | 0.679 | 12224 | 0.495 | 12534 | 0.409 | 13344 | 0.348 |
| 11415 | 0.558 | 12225 | 0.375 | 12535 | 0.289 | 13345 | 0.228 |

| Health  state | Utility value | Health  state | Utility value | Health state | Utility value | Health  state | Utility value |
| --- | --- | --- | --- | --- | --- | --- | --- |
| 13351 | 0.441 | 14211 | 0.724 | 14521 | 0.586 | 15331 | 0.537 |
| 13352 | 0.361 | 14212 | 0.644 | 14522 | 0.506 | 15332 | 0.457 |
| 13353 | 0.340 | 14213 | 0.623 | 14523 | 0.485 | 15333 | 0.436 |
| 13354 | 0.224 | 14214 | 0.507 | 14524 | 0.369 | 15334 | 0.320 |
| 13355 | 0.104 | 14215 | 0.387 | 14525 | 0.249 | 15335 | 0.200 |
| 13411 | 0.716 | 14221 | 0.655 | 14531 | 0.569 | 15341 | 0.454 |
| 13412 | 0.636 | 14222 | 0.575 | 14532 | 0.489 | 15342 | 0.374 |
| 13413 | 0.616 | 14223 | 0.554 | 14533 | 0.468 | 15343 | 0.354 |
| 13414 | 0.500 | 14224 | 0.438 | 14534 | 0.352 | 15344 | 0.238 |
| 13415 | 0.379 | 14225 | 0.318 | 14535 | 0.232 | 15345 | 0.117 |
| 13421 | 0.648 | 14231 | 0.638 | 14541 | 0.486 | 15351 | 0.330 |
| 13422 | 0.568 | 14232 | 0.558 | 14542 | 0.406 | 15352 | 0.250 |
| 13423 | 0.547 | 14233 | 0.537 | 14543 | 0.386 | 15353 | 0.230 |
| 13424 | 0.431 | 14234 | 0.421 | 14544 | 0.270 | 15354 | 0.114 |
| 13425 | 0.311 | 14235 | 0.301 | 14545 | 0.149 | 15355 | -0.007 |
| 13431 | 0.630 | 14241 | 0.555 | 14551 | 0.362 | 15411 | 0.606 |
| 13432 | 0.551 | 14242 | 0.476 | 14552 | 0.282 | 15412 | 0.526 |
| 13433 | 0.530 | 14243 | 0.455 | 14553 | 0.262 | 15413 | 0.505 |
| 13434 | 0.414 | 14244 | 0.339 | 14554 | 0.146 | 15414 | 0.389 |
| 13435 | 0.293 | 14245 | 0.218 | 14555 | 0.025 | 15415 | 0.269 |
| 13441 | 0.548 | 14251 | 0.432 | 15111 | 0.710 | 15421 | 0.537 |
| 13442 | 0.468 | 14252 | 0.352 | 15112 | 0.631 | 15422 | 0.457 |
| 13443 | 0.447 | 14253 | 0.331 | 15113 | 0.610 | 15423 | 0.436 |
| 13444 | 0.331 | 14254 | 0.215 | 15114 | 0.494 | 15424 | 0.320 |
| 13445 | 0.211 | 14255 | 0.095 | 15115 | 0.373 | 15425 | 0.200 |
| 13451 | 0.424 | 14311 | 0.706 | 15121 | 0.642 | 15431 | 0.520 |
| 13452 | 0.344 | 14312 | 0.627 | 15122 | 0.562 | 15432 | 0.440 |
| 13453 | 0.323 | 14313 | 0.606 | 15123 | 0.541 | 15433 | 0.419 |
| 13454 | 0.207 | 14314 | 0.490 | 15124 | 0.425 | 15434 | 0.303 |
| 13455 | 0.087 | 14315 | 0.369 | 15125 | 0.305 | 15435 | 0.183 |
| 13511 | 0.682 | 14321 | 0.638 | 15131 | 0.625 | 15441 | 0.437 |
| 13512 | 0.602 | 14322 | 0.558 | 15132 | 0.545 | 15442 | 0.357 |
| 13513 | 0.581 | 14323 | 0.537 | 15133 | 0.524 | 15443 | 0.337 |
| 13514 | 0.465 | 14324 | 0.421 | 15134 | 0.408 | 15444 | 0.221 |
| 13515 | 0.345 | 14325 | 0.301 | 15135 | 0.288 | 15445 | 0.100 |
| 13521 | 0.613 | 14331 | 0.621 | 15141 | 0.542 | 15451 | 0.313 |
| 13522 | 0.533 | 14332 | 0.541 | 15142 | 0.462 | 15452 | 0.233 |
| 13523 | 0.512 | 14333 | 0.520 | 15143 | 0.441 | 15453 | 0.213 |
| 13524 | 0.396 | 14334 | 0.404 | 15144 | 0.325 | 15454 | 0.097 |
| 13525 | 0.276 | 14335 | 0.284 | 15145 | 0.205 | 15455 | -0.024 |
| 13531 | 0.596 | 14341 | 0.538 | 15151 | 0.418 | 15511 | 0.571 |
| 13532 | 0.516 | 14342 | 0.458 | 15152 | 0.338 | 15512 | 0.491 |
| 13533 | 0.495 | 14343 | 0.437 | 15153 | 0.317 | 15513 | 0.470 |
| 13534 | 0.379 | 14344 | 0.321 | 15154 | 0.201 | 15514 | 0.354 |
| 13535 | 0.259 | 14345 | 0.201 | 15155 | 0.081 | 15515 | 0.234 |
| 13541 | 0.513 | 14351 | 0.414 | 15211 | 0.640 | 15521 | 0.502 |
| 13542 | 0.433 | 14352 | 0.334 | 15212 | 0.560 | 15522 | 0.422 |
| 13543 | 0.413 | 14353 | 0.313 | 15213 | 0.539 | 15523 | 0.401 |
| 13544 | 0.297 | 14354 | 0.198 | 15214 | 0.423 | 15524 | 0.285 |
| 13545 | 0.176 | 14355 | 0.077 | 15215 | 0.303 | 15525 | 0.165 |
| 13551 | 0.389 | 14411 | 0.689 | 15221 | 0.571 | 15531 | 0.485 |
| 13552 | 0.309 | 14412 | 0.610 | 15222 | 0.491 | 15532 | 0.405 |
| 13553 | 0.289 | 14413 | 0.589 | 15223 | 0.470 | 15533 | 0.384 |
| 13554 | 0.173 | 14414 | 0.473 | 15224 | 0.355 | 15534 | 0.268 |
| 13555 | 0.052 | 14415 | 0.352 | 15225 | 0.234 | 15535 | 0.148 |
| 14111 | 0.794 | 14421 | 0.621 | 15231 | 0.554 | 15541 | 0.402 |
| 14112 | 0.714 | 14422 | 0.541 | 15232 | 0.474 | 15542 | 0.323 |
| 14113 | 0.694 | 14423 | 0.520 | 15233 | 0.453 | 15543 | 0.302 |
| 14114 | 0.578 | 14424 | 0.404 | 15234 | 0.337 | 15544 | 0.186 |
| 14115 | 0.457 | 14425 | 0.284 | 15235 | 0.217 | 15545 | 0.065 |
| 14121 | 0.725 | 14431 | 0.604 | 15241 | 0.472 | 15551 | 0.279 |
| 14122 | 0.646 | 14432 | 0.524 | 15242 | 0.392 | 15552 | 0.199 |
| 14123 | 0.625 | 14433 | 0.503 | 15243 | 0.371 | 15553 | 0.178 |
| 14124 | 0.509 | 14434 | 0.387 | 15244 | 0.255 | 15554 | 0.062 |
| 14125 | 0.388 | 14435 | 0.267 | 15245 | 0.135 | 15555 | -0.059 |
| 14131 | 0.708 | 14441 | 0.521 | 15251 | 0.348 | 21111 | 0.901 |
| 14132 | 0.628 | 14442 | 0.441 | 15252 | 0.268 | 21112 | 0.821 |
| 14133 | 0.608 | 14443 | 0.420 | 15253 | 0.247 | 21113 | 0.800 |
| 14134 | 0.492 | 14444 | 0.304 | 15254 | 0.131 | 21114 | 0.684 |
| 14135 | 0.371 | 14445 | 0.184 | 15255 | 0.011 | 21115 | 0.564 |
| 14141 | 0.626 | 14451 | 0.397 | 15311 | 0.623 | 21121 | 0.832 |
| 14142 | 0.546 | 14452 | 0.317 | 15312 | 0.543 | 21122 | 0.752 |
| 14143 | 0.525 | 14453 | 0.296 | 15313 | 0.522 | 21123 | 0.731 |
| 14144 | 0.409 | 14454 | 0.181 | 15314 | 0.406 | 21124 | 0.615 |
| 14145 | 0.289 | 14455 | 0.060 | 15315 | 0.286 | 21125 | 0.495 |
| 14151 | 0.502 | 14511 | 0.655 | 15321 | 0.554 | 21131 | 0.815 |
| 14152 | 0.422 | 14512 | 0.575 | 15322 | 0.474 | 21132 | 0.735 |
| 14153 | 0.401 | 14513 | 0.554 | 15323 | 0.453 | 21133 | 0.714 |
| 14154 | 0.285 | 14514 | 0.438 | 15324 | 0.337 | 21134 | 0.598 |
| 14155 | 0.165 | 14515 | 0.318 | 15325 | 0.217 | 21135 | 0.478 |

| Health  state | Utility value | Health  state | Utility value | Health state | Utility value | Health  state | Utility value |
| --- | --- | --- | --- | --- | --- | --- | --- |
| 21141 | 0.732 | 21451 | 0.504 | 22311 | 0.664 | 23121 | 0.653 |
| 21142 | 0.652 | 21452 | 0.424 | 22312 | 0.584 | 23122 | 0.573 |
| 21143 | 0.632 | 21453 | 0.403 | 22313 | 0.563 | 23123 | 0.552 |
| 21144 | 0.516 | 21454 | 0.287 | 22314 | 0.447 | 23124 | 0.436 |
| 21145 | 0.395 | 21455 | 0.167 | 22315 | 0.327 | 23125 | 0.316 |
| 21151 | 0.608 | 21511 | 0.761 | 22321 | 0.595 | 23131 | 0.636 |
| 21152 | 0.528 | 21512 | 0.681 | 22322 | 0.515 | 23132 | 0.556 |
| 21153 | 0.508 | 21513 | 0.660 | 22323 | 0.494 | 23133 | 0.535 |
| 21154 | 0.392 | 21514 | 0.544 | 22324 | 0.378 | 23134 | 0.419 |
| 21155 | 0.271 | 21515 | 0.424 | 22325 | 0.258 | 23135 | 0.299 |
| 21211 | 0.830 | 21521 | 0.692 | 22331 | 0.578 | 23141 | 0.553 |
| 21212 | 0.750 | 21522 | 0.612 | 22332 | 0.498 | 23142 | 0.474 |
| 21213 | 0.729 | 21523 | 0.592 | 22333 | 0.477 | 23143 | 0.453 |
| 21214 | 0.614 | 21524 | 0.476 | 22334 | 0.361 | 23144 | 0.337 |
| 21215 | 0.493 | 21525 | 0.355 | 22335 | 0.241 | 23145 | 0.216 |
| 21221 | 0.761 | 21531 | 0.675 | 22341 | 0.496 | 23151 | 0.430 |
| 21222 | 0.681 | 21532 | 0.595 | 22342 | 0.416 | 23152 | 0.350 |
| 21223 | 0.661 | 21533 | 0.574 | 22343 | 0.395 | 23153 | 0.329 |
| 21224 | 0.545 | 21534 | 0.458 | 22344 | 0.279 | 23154 | 0.213 |
| 21225 | 0.424 | 21535 | 0.338 | 22345 | 0.159 | 23155 | 0.093 |
| 21231 | 0.744 | 21541 | 0.593 | 22351 | 0.372 | 23211 | 0.651 |
| 21232 | 0.664 | 21542 | 0.513 | 22352 | 0.292 | 23212 | 0.571 |
| 21233 | 0.644 | 21543 | 0.492 | 22353 | 0.271 | 23213 | 0.551 |
| 21234 | 0.528 | 21544 | 0.376 | 22354 | 0.155 | 23214 | 0.435 |
| 21235 | 0.407 | 21545 | 0.256 | 22355 | 0.035 | 23215 | 0.314 |
| 21241 | 0.662 | 21551 | 0.469 | 22411 | 0.647 | 23221 | 0.583 |
| 21242 | 0.582 | 21552 | 0.389 | 22412 | 0.567 | 23222 | 0.503 |
| 21243 | 0.561 | 21553 | 0.368 | 22413 | 0.546 | 23223 | 0.482 |
| 21244 | 0.445 | 21554 | 0.252 | 22414 | 0.430 | 23224 | 0.366 |
| 21245 | 0.325 | 21555 | 0.132 | 22415 | 0.310 | 23225 | 0.246 |
| 21251 | 0.538 | 22111 | 0.752 | 22421 | 0.578 | 23231 | 0.565 |
| 21252 | 0.458 | 22112 | 0.672 | 22422 | 0.498 | 23232 | 0.486 |
| 21253 | 0.437 | 22113 | 0.651 | 22423 | 0.477 | 23233 | 0.465 |
| 21254 | 0.321 | 22114 | 0.535 | 22424 | 0.361 | 23234 | 0.349 |
| 21255 | 0.201 | 22115 | 0.415 | 22425 | 0.241 | 23235 | 0.228 |
| 21311 | 0.813 | 22121 | 0.683 | 22431 | 0.561 | 23241 | 0.483 |
| 21312 | 0.733 | 22122 | 0.603 | 22432 | 0.481 | 23242 | 0.403 |
| 21313 | 0.712 | 22123 | 0.582 | 22433 | 0.460 | 23243 | 0.382 |
| 21314 | 0.596 | 22124 | 0.466 | 22434 | 0.344 | 23244 | 0.266 |
| 21315 | 0.476 | 22125 | 0.346 | 22435 | 0.224 | 23245 | 0.146 |
| 21321 | 0.744 | 22131 | 0.666 | 22441 | 0.479 | 23251 | 0.359 |
| 21322 | 0.664 | 22132 | 0.586 | 22442 | 0.399 | 23252 | 0.279 |
| 21323 | 0.643 | 22133 | 0.565 | 22443 | 0.378 | 23253 | 0.258 |
| 21324 | 0.527 | 22134 | 0.449 | 22444 | 0.262 | 23254 | 0.142 |
| 21325 | 0.407 | 22135 | 0.329 | 22445 | 0.142 | 23255 | 0.022 |
| 21331 | 0.727 | 22141 | 0.583 | 22451 | 0.355 | 23311 | 0.634 |
| 21332 | 0.647 | 22142 | 0.503 | 22452 | 0.275 | 23312 | 0.554 |
| 21333 | 0.626 | 22143 | 0.483 | 22453 | 0.254 | 23313 | 0.533 |
| 21334 | 0.510 | 22144 | 0.367 | 22454 | 0.138 | 23314 | 0.417 |
| 21335 | 0.390 | 22145 | 0.246 | 22455 | 0.018 | 23315 | 0.297 |
| 21341 | 0.644 | 22151 | 0.459 | 22511 | 0.612 | 23321 | 0.565 |
| 21342 | 0.565 | 22152 | 0.380 | 22512 | 0.532 | 23322 | 0.485 |
| 21343 | 0.544 | 22153 | 0.359 | 22513 | 0.511 | 23323 | 0.465 |
| 21344 | 0.428 | 22154 | 0.243 | 22514 | 0.395 | 23324 | 0.349 |
| 21345 | 0.307 | 22155 | 0.122 | 22515 | 0.275 | 23325 | 0.228 |
| 21351 | 0.521 | 22211 | 0.681 | 22521 | 0.543 | 23331 | 0.548 |
| 21352 | 0.441 | 22212 | 0.601 | 22522 | 0.463 | 23332 | 0.468 |
| 21353 | 0.420 | 22213 | 0.581 | 22523 | 0.443 | 23333 | 0.447 |
| 21354 | 0.304 | 22214 | 0.465 | 22524 | 0.327 | 23334 | 0.331 |
| 21355 | 0.184 | 22215 | 0.344 | 22525 | 0.206 | 23335 | 0.211 |
| 21411 | 0.796 | 22221 | 0.612 | 22531 | 0.526 | 23341 | 0.466 |
| 21412 | 0.716 | 22222 | 0.533 | 22532 | 0.446 | 23342 | 0.386 |
| 21413 | 0.695 | 22223 | 0.512 | 22533 | 0.426 | 23343 | 0.365 |
| 21414 | 0.579 | 22224 | 0.396 | 22534 | 0.310 | 23344 | 0.249 |
| 21415 | 0.459 | 22225 | 0.275 | 22535 | 0.189 | 23345 | 0.129 |
| 21421 | 0.727 | 22231 | 0.595 | 22541 | 0.444 | 23351 | 0.342 |
| 21422 | 0.647 | 22232 | 0.516 | 22542 | 0.364 | 23352 | 0.262 |
| 21423 | 0.626 | 22233 | 0.495 | 22543 | 0.343 | 23353 | 0.241 |
| 21424 | 0.510 | 22234 | 0.379 | 22544 | 0.227 | 23354 | 0.125 |
| 21425 | 0.390 | 22235 | 0.258 | 22545 | 0.107 | 23355 | 0.005 |
| 21431 | 0.710 | 22241 | 0.513 | 22551 | 0.320 | 23411 | 0.617 |
| 21432 | 0.630 | 22242 | 0.433 | 22552 | 0.240 | 23412 | 0.537 |
| 21433 | 0.609 | 22243 | 0.412 | 22553 | 0.219 | 23413 | 0.516 |
| 21434 | 0.493 | 22244 | 0.296 | 22554 | 0.103 | 23414 | 0.400 |
| 21435 | 0.373 | 22245 | 0.176 | 22555 | -0.017 | 23415 | 0.280 |
| 21441 | 0.627 | 22251 | 0.389 | 23111 | 0.722 | 23421 | 0.548 |
| 21442 | 0.548 | 22252 | 0.309 | 23112 | 0.642 | 23422 | 0.468 |
| 21443 | 0.527 | 22253 | 0.288 | 23113 | 0.621 | 23423 | 0.447 |
| 21444 | 0.411 | 22254 | 0.172 | 23114 | 0.505 | 23424 | 0.332 |
| 21445 | 0.290 | 22255 | 0.052 | 23115 | 0.385 | 23425 | 0.211 |

| Health  state | Utility value | Health  state | Utility value | Health state | Utility value | Health  state | Utility value |
| --- | --- | --- | --- | --- | --- | --- | --- |
| 23431 | 0.531 | 24241 | 0.456 | 24551 | 0.263 | 25411 | 0.506 |
| 23432 | 0.451 | 24242 | 0.376 | 24552 | 0.183 | 25412 | 0.426 |
| 23433 | 0.430 | 24243 | 0.355 | 24553 | 0.162 | 25413 | 0.406 |
| 23434 | 0.314 | 24244 | 0.239 | 24554 | 0.046 | 25414 | 0.290 |
| 23435 | 0.194 | 24245 | 0.119 | 24555 | -0.074 | 25415 | 0.169 |
| 23441 | 0.449 | 24251 | 0.332 | 25111 | 0.611 | 25421 | 0.437 |
| 23442 | 0.369 | 24252 | 0.252 | 25112 | 0.531 | 25422 | 0.357 |
| 23443 | 0.348 | 24253 | 0.231 | 25113 | 0.510 | 25423 | 0.337 |
| 23444 | 0.232 | 24254 | 0.116 | 25114 | 0.394 | 25424 | 0.221 |
| 23445 | 0.112 | 24255 | -0.005 | 25115 | 0.274 | 25425 | 0.100 |
| 23451 | 0.325 | 24311 | 0.607 | 25121 | 0.542 | 25431 | 0.420 |
| 23452 | 0.245 | 24312 | 0.527 | 25122 | 0.462 | 25432 | 0.340 |
| 23453 | 0.224 | 24313 | 0.506 | 25123 | 0.442 | 25433 | 0.320 |
| 23454 | 0.108 | 24314 | 0.390 | 25124 | 0.326 | 25434 | 0.204 |
| 23455 | -0.012 | 24315 | 0.270 | 25125 | 0.205 | 25435 | 0.083 |
| 23511 | 0.582 | 24321 | 0.538 | 25131 | 0.525 | 25441 | 0.338 |
| 23512 | 0.502 | 24322 | 0.458 | 25132 | 0.445 | 25442 | 0.258 |
| 23513 | 0.482 | 24323 | 0.438 | 25133 | 0.424 | 25443 | 0.237 |
| 23514 | 0.366 | 24324 | 0.322 | 25134 | 0.308 | 25444 | 0.121 |
| 23515 | 0.245 | 24325 | 0.201 | 25135 | 0.188 | 25445 | 0.001 |
| 23521 | 0.513 | 24331 | 0.521 | 25141 | 0.443 | 25451 | 0.214 |
| 23522 | 0.434 | 24332 | 0.441 | 25142 | 0.363 | 25452 | 0.134 |
| 23523 | 0.413 | 24333 | 0.420 | 25143 | 0.342 | 25453 | 0.113 |
| 23524 | 0.297 | 24334 | 0.305 | 25144 | 0.226 | 25454 | -0.003 |
| 23525 | 0.176 | 24335 | 0.184 | 25145 | 0.106 | 25455 | -0.123 |
| 23531 | 0.496 | 24341 | 0.439 | 25151 | 0.319 | 25511 | 0.471 |
| 23532 | 0.416 | 24342 | 0.359 | 25152 | 0.239 | 25512 | 0.392 |
| 23533 | 0.396 | 24343 | 0.338 | 25153 | 0.218 | 25513 | 0.371 |
| 23534 | 0.280 | 24344 | 0.222 | 25154 | 0.102 | 25514 | 0.255 |
| 23535 | 0.159 | 24345 | 0.102 | 25155 | -0.018 | 25515 | 0.134 |
| 23541 | 0.414 | 24351 | 0.315 | 25211 | 0.541 | 25521 | 0.403 |
| 23542 | 0.334 | 24352 | 0.235 | 25212 | 0.461 | 25522 | 0.323 |
| 23543 | 0.313 | 24353 | 0.214 | 25213 | 0.440 | 25523 | 0.302 |
| 23544 | 0.197 | 24354 | 0.098 | 25214 | 0.324 | 25524 | 0.186 |
| 23545 | 0.077 | 24355 | -0.022 | 25215 | 0.204 | 25525 | 0.066 |
| 23551 | 0.290 | 24411 | 0.590 | 25221 | 0.472 | 25531 | 0.386 |
| 23552 | 0.210 | 24412 | 0.510 | 25222 | 0.392 | 25532 | 0.306 |
| 23553 | 0.189 | 24413 | 0.489 | 25223 | 0.371 | 25533 | 0.285 |
| 23554 | 0.073 | 24414 | 0.373 | 25224 | 0.255 | 25534 | 0.169 |
| 23555 | -0.047 | 24415 | 0.253 | 25225 | 0.135 | 25535 | 0.049 |
| 24111 | 0.695 | 24421 | 0.521 | 25231 | 0.455 | 25541 | 0.303 |
| 24112 | 0.615 | 24422 | 0.441 | 25232 | 0.375 | 25542 | 0.223 |
| 24113 | 0.594 | 24423 | 0.421 | 25233 | 0.354 | 25543 | 0.202 |
| 24114 | 0.478 | 24424 | 0.305 | 25234 | 0.238 | 25544 | 0.086 |
| 24115 | 0.358 | 24425 | 0.184 | 25235 | 0.118 | 25545 | -0.034 |
| 24121 | 0.626 | 24431 | 0.504 | 25241 | 0.372 | 25551 | 0.179 |
| 24122 | 0.546 | 24432 | 0.424 | 25242 | 0.292 | 25552 | 0.099 |
| 24123 | 0.525 | 24433 | 0.403 | 25243 | 0.272 | 25553 | 0.078 |
| 24124 | 0.409 | 24434 | 0.288 | 25244 | 0.156 | 25554 | -0.038 |
| 24125 | 0.289 | 24435 | 0.167 | 25245 | 0.035 | 25555 | -0.158 |
| 24131 | 0.609 | 24441 | 0.422 | 25251 | 0.248 | 31111 | 0.879 |
| 24132 | 0.529 | 24442 | 0.342 | 25252 | 0.168 | 31112 | 0.799 |
| 24133 | 0.508 | 24443 | 0.321 | 25253 | 0.148 | 31113 | 0.779 |
| 24134 | 0.392 | 24444 | 0.205 | 25254 | 0.032 | 31114 | 0.663 |
| 24135 | 0.272 | 24445 | 0.085 | 25255 | -0.089 | 31115 | 0.542 |
| 24141 | 0.527 | 24451 | 0.298 | 25311 | 0.523 | 31121 | 0.810 |
| 24142 | 0.447 | 24452 | 0.218 | 25312 | 0.443 | 31122 | 0.730 |
| 24143 | 0.426 | 24453 | 0.197 | 25313 | 0.423 | 31123 | 0.710 |
| 24144 | 0.310 | 24454 | 0.081 | 25314 | 0.307 | 31124 | 0.594 |
| 24145 | 0.190 | 24455 | -0.039 | 25315 | 0.186 | 31125 | 0.473 |
| 24151 | 0.403 | 24511 | 0.555 | 25321 | 0.454 | 31131 | 0.793 |
| 24152 | 0.323 | 24512 | 0.475 | 25322 | 0.375 | 31132 | 0.713 |
| 24153 | 0.302 | 24513 | 0.455 | 25323 | 0.354 | 31133 | 0.693 |
| 24154 | 0.186 | 24514 | 0.339 | 25324 | 0.238 | 31134 | 0.577 |
| 24155 | 0.066 | 24515 | 0.218 | 25325 | 0.117 | 31135 | 0.456 |
| 24211 | 0.624 | 24521 | 0.486 | 25331 | 0.437 | 31141 | 0.711 |
| 24212 | 0.545 | 24522 | 0.407 | 25332 | 0.357 | 31142 | 0.631 |
| 24213 | 0.524 | 24523 | 0.386 | 25333 | 0.337 | 31143 | 0.610 |
| 24214 | 0.408 | 24524 | 0.270 | 25334 | 0.221 | 31144 | 0.494 |
| 24215 | 0.287 | 24525 | 0.149 | 25335 | 0.100 | 31145 | 0.374 |
| 24221 | 0.556 | 24531 | 0.469 | 25341 | 0.355 | 31151 | 0.587 |
| 24222 | 0.476 | 24532 | 0.389 | 25342 | 0.275 | 31152 | 0.507 |
| 24223 | 0.455 | 24533 | 0.369 | 25343 | 0.254 | 31153 | 0.486 |
| 24224 | 0.339 | 24534 | 0.253 | 25344 | 0.138 | 31154 | 0.370 |
| 24225 | 0.219 | 24535 | 0.132 | 25345 | 0.018 | 31155 | 0.250 |
| 24231 | 0.539 | 24541 | 0.387 | 25351 | 0.231 | 31211 | 0.809 |
| 24232 | 0.459 | 24542 | 0.307 | 25352 | 0.151 | 31212 | 0.729 |
| 24233 | 0.438 | 24543 | 0.286 | 25353 | 0.130 | 31213 | 0.708 |
| 24234 | 0.322 | 24544 | 0.170 | 25354 | 0.014 | 31214 | 0.592 |
| 24235 | 0.202 | 24545 | 0.050 | 25355 | -0.106 | 31215 | 0.472 |

| Health  state | Utility value | Health  state | Utility value | Health state | Utility value | Health  state | Utility value |
| --- | --- | --- | --- | --- | --- | --- | --- |
| 31221 | 0.740 | 31531 | 0.654 | 32341 | 0.474 | 33151 | 0.408 |
| 31222 | 0.660 | 31532 | 0.574 | 32342 | 0.394 | 33152 | 0.328 |
| 31223 | 0.639 | 31533 | 0.553 | 32343 | 0.373 | 33153 | 0.307 |
| 31224 | 0.523 | 31534 | 0.437 | 32344 | 0.258 | 33154 | 0.191 |
| 31225 | 0.403 | 31535 | 0.317 | 32345 | 0.137 | 33155 | 0.071 |
| 31231 | 0.723 | 31541 | 0.571 | 32351 | 0.350 | 33211 | 0.630 |
| 31232 | 0.643 | 31542 | 0.491 | 32352 | 0.270 | 33212 | 0.550 |
| 31233 | 0.622 | 31543 | 0.471 | 32353 | 0.250 | 33213 | 0.529 |
| 31234 | 0.506 | 31544 | 0.355 | 32354 | 0.134 | 33214 | 0.413 |
| 31235 | 0.386 | 31545 | 0.234 | 32355 | 0.013 | 33215 | 0.293 |
| 31241 | 0.640 | 31551 | 0.447 | 32411 | 0.626 | 33221 | 0.561 |
| 31242 | 0.561 | 31552 | 0.367 | 32412 | 0.546 | 33222 | 0.481 |
| 31243 | 0.540 | 31553 | 0.347 | 32413 | 0.525 | 33223 | 0.460 |
| 31244 | 0.424 | 31554 | 0.231 | 32414 | 0.409 | 33224 | 0.345 |
| 31245 | 0.303 | 31555 | 0.110 | 32415 | 0.289 | 33225 | 0.224 |
| 31251 | 0.516 | 32111 | 0.730 | 32421 | 0.557 | 33231 | 0.544 |
| 31252 | 0.437 | 32112 | 0.650 | 32422 | 0.477 | 33232 | 0.464 |
| 31253 | 0.416 | 32113 | 0.630 | 32423 | 0.456 | 33233 | 0.443 |
| 31254 | 0.300 | 32114 | 0.514 | 32424 | 0.340 | 33234 | 0.327 |
| 31255 | 0.179 | 32115 | 0.393 | 32425 | 0.220 | 33235 | 0.207 |
| 31311 | 0.791 | 32121 | 0.662 | 32431 | 0.540 | 33241 | 0.462 |
| 31312 | 0.712 | 32122 | 0.582 | 32432 | 0.460 | 33242 | 0.382 |
| 31313 | 0.691 | 32123 | 0.561 | 32433 | 0.439 | 33243 | 0.361 |
| 31314 | 0.575 | 32124 | 0.445 | 32434 | 0.323 | 33244 | 0.245 |
| 31315 | 0.454 | 32125 | 0.325 | 32435 | 0.203 | 33245 | 0.125 |
| 31321 | 0.723 | 32131 | 0.644 | 32441 | 0.457 | 33251 | 0.338 |
| 31322 | 0.643 | 32132 | 0.565 | 32442 | 0.377 | 33252 | 0.258 |
| 31323 | 0.622 | 32133 | 0.544 | 32443 | 0.356 | 33253 | 0.237 |
| 31324 | 0.506 | 32134 | 0.428 | 32444 | 0.241 | 33254 | 0.121 |
| 31325 | 0.386 | 32135 | 0.307 | 32445 | 0.120 | 33255 | 0.001 |
| 31331 | 0.706 | 32141 | 0.562 | 32451 | 0.333 | 33311 | 0.613 |
| 31332 | 0.626 | 32142 | 0.482 | 32452 | 0.253 | 33312 | 0.533 |
| 31333 | 0.605 | 32143 | 0.461 | 32453 | 0.233 | 33313 | 0.512 |
| 31334 | 0.489 | 32144 | 0.345 | 32454 | 0.117 | 33314 | 0.396 |
| 31335 | 0.369 | 32145 | 0.225 | 32455 | -0.004 | 33315 | 0.276 |
| 31341 | 0.623 | 32151 | 0.438 | 32511 | 0.591 | 33321 | 0.544 |
| 31342 | 0.543 | 32152 | 0.358 | 32512 | 0.511 | 33322 | 0.464 |
| 31343 | 0.522 | 32153 | 0.337 | 32513 | 0.490 | 33323 | 0.443 |
| 31344 | 0.406 | 32154 | 0.221 | 32514 | 0.374 | 33324 | 0.327 |
| 31345 | 0.286 | 32155 | 0.101 | 32515 | 0.254 | 33325 | 0.207 |
| 31351 | 0.499 | 32211 | 0.660 | 32521 | 0.522 | 33331 | 0.527 |
| 31352 | 0.419 | 32212 | 0.580 | 32522 | 0.442 | 33332 | 0.447 |
| 31353 | 0.398 | 32213 | 0.559 | 32523 | 0.421 | 33333 | 0.426 |
| 31354 | 0.282 | 32214 | 0.443 | 32524 | 0.305 | 33334 | 0.310 |
| 31355 | 0.162 | 32215 | 0.323 | 32525 | 0.185 | 33335 | 0.190 |
| 31411 | 0.774 | 32221 | 0.591 | 32531 | 0.505 | 33341 | 0.444 |
| 31412 | 0.694 | 32222 | 0.511 | 32532 | 0.425 | 33342 | 0.364 |
| 31413 | 0.674 | 32223 | 0.490 | 32533 | 0.404 | 33343 | 0.344 |
| 31414 | 0.558 | 32224 | 0.374 | 32534 | 0.288 | 33344 | 0.228 |
| 31415 | 0.437 | 32225 | 0.254 | 32535 | 0.168 | 33345 | 0.107 |
| 31421 | 0.706 | 32231 | 0.574 | 32541 | 0.422 | 33351 | 0.320 |
| 31422 | 0.626 | 32232 | 0.494 | 32542 | 0.342 | 33352 | 0.240 |
| 31423 | 0.605 | 32233 | 0.473 | 32543 | 0.322 | 33353 | 0.220 |
| 31424 | 0.489 | 32234 | 0.357 | 32544 | 0.206 | 33354 | 0.104 |
| 31425 | 0.369 | 32235 | 0.237 | 32545 | 0.085 | 33355 | -0.017 |
| 31431 | 0.688 | 32241 | 0.492 | 32551 | 0.298 | 33411 | 0.596 |
| 31432 | 0.609 | 32242 | 0.412 | 32552 | 0.219 | 33412 | 0.516 |
| 31433 | 0.588 | 32243 | 0.391 | 32553 | 0.198 | 33413 | 0.495 |
| 31434 | 0.472 | 32244 | 0.275 | 32554 | 0.082 | 33414 | 0.379 |
| 31435 | 0.351 | 32245 | 0.155 | 32555 | -0.039 | 33415 | 0.259 |
| 31441 | 0.606 | 32251 | 0.368 | 33111 | 0.700 | 33421 | 0.527 |
| 31442 | 0.526 | 32252 | 0.288 | 33112 | 0.621 | 33422 | 0.447 |
| 31443 | 0.505 | 32253 | 0.267 | 33113 | 0.600 | 33423 | 0.426 |
| 31444 | 0.389 | 32254 | 0.151 | 33114 | 0.484 | 33424 | 0.310 |
| 31445 | 0.269 | 32255 | 0.031 | 33115 | 0.363 | 33425 | 0.190 |
| 31451 | 0.482 | 32311 | 0.643 | 33121 | 0.632 | 33431 | 0.510 |
| 31452 | 0.402 | 32312 | 0.563 | 33122 | 0.552 | 33432 | 0.430 |
| 31453 | 0.381 | 32313 | 0.542 | 33123 | 0.531 | 33433 | 0.409 |
| 31454 | 0.265 | 32314 | 0.426 | 33124 | 0.415 | 33434 | 0.293 |
| 31455 | 0.145 | 32315 | 0.306 | 33125 | 0.295 | 33435 | 0.173 |
| 31511 | 0.740 | 32321 | 0.574 | 33131 | 0.615 | 33441 | 0.427 |
| 31512 | 0.660 | 32322 | 0.494 | 33132 | 0.535 | 33442 | 0.347 |
| 31513 | 0.639 | 32323 | 0.473 | 33133 | 0.514 | 33443 | 0.327 |
| 31514 | 0.523 | 32324 | 0.357 | 33134 | 0.398 | 33444 | 0.211 |
| 31515 | 0.403 | 32325 | 0.237 | 33135 | 0.278 | 33445 | 0.090 |
| 31521 | 0.671 | 32331 | 0.557 | 33141 | 0.532 | 33451 | 0.303 |
| 31522 | 0.591 | 32332 | 0.477 | 33142 | 0.452 | 33452 | 0.223 |
| 31523 | 0.570 | 32333 | 0.456 | 33143 | 0.431 | 33453 | 0.203 |
| 31524 | 0.454 | 32334 | 0.340 | 33144 | 0.315 | 33454 | 0.087 |
| 31525 | 0.334 | 32335 | 0.220 | 33145 | 0.195 | 33455 | -0.034 |

| Health  state | Utility value | Health  state | Utility value | Health state | Utility value | Health  state | Utility value |
| --- | --- | --- | --- | --- | --- | --- | --- |
| 33511 | 0.561 | 34321 | 0.517 | 35131 | 0.504 | 35441 | 0.316 |
| 33512 | 0.481 | 34322 | 0.437 | 35132 | 0.424 | 35442 | 0.237 |
| 33513 | 0.460 | 34323 | 0.416 | 35133 | 0.403 | 35443 | 0.216 |
| 33514 | 0.344 | 34324 | 0.300 | 35134 | 0.287 | 35444 | 0.100 |
| 33515 | 0.224 | 34325 | 0.180 | 35135 | 0.167 | 35445 | -0.021 |
| 33521 | 0.492 | 34331 | 0.500 | 35141 | 0.421 | 35451 | 0.192 |
| 33522 | 0.412 | 34332 | 0.420 | 35142 | 0.341 | 35452 | 0.113 |
| 33523 | 0.391 | 34333 | 0.399 | 35143 | 0.321 | 35453 | 0.092 |
| 33524 | 0.275 | 34334 | 0.283 | 35144 | 0.205 | 35454 | -0.024 |
| 33525 | 0.155 | 34335 | 0.163 | 35145 | 0.084 | 35455 | -0.145 |
| 33531 | 0.475 | 34341 | 0.417 | 35151 | 0.297 | 35511 | 0.450 |
| 33532 | 0.395 | 34342 | 0.337 | 35152 | 0.217 | 35512 | 0.370 |
| 33533 | 0.374 | 34343 | 0.317 | 35153 | 0.197 | 35513 | 0.349 |
| 33534 | 0.258 | 34344 | 0.201 | 35154 | 0.081 | 35514 | 0.233 |
| 33535 | 0.138 | 34345 | 0.080 | 35155 | -0.040 | 35515 | 0.113 |
| 33541 | 0.392 | 34351 | 0.293 | 35211 | 0.519 | 35521 | 0.381 |
| 33542 | 0.313 | 34352 | 0.213 | 35212 | 0.439 | 35522 | 0.301 |
| 33543 | 0.292 | 34353 | 0.193 | 35213 | 0.418 | 35523 | 0.280 |
| 33544 | 0.176 | 34354 | 0.077 | 35214 | 0.303 | 35524 | 0.165 |
| 33545 | 0.055 | 34355 | -0.044 | 35215 | 0.182 | 35525 | 0.044 |
| 33551 | 0.269 | 34411 | 0.569 | 35221 | 0.450 | 35531 | 0.364 |
| 33552 | 0.189 | 34412 | 0.489 | 35222 | 0.370 | 35532 | 0.284 |
| 33553 | 0.168 | 34413 | 0.468 | 35223 | 0.350 | 35533 | 0.263 |
| 33554 | 0.052 | 34414 | 0.352 | 35224 | 0.234 | 35534 | 0.147 |
| 33555 | -0.069 | 34415 | 0.232 | 35225 | 0.113 | 35535 | 0.027 |
| 34111 | 0.673 | 34421 | 0.500 | 35231 | 0.433 | 35541 | 0.282 |
| 34112 | 0.594 | 34422 | 0.420 | 35232 | 0.353 | 35542 | 0.202 |
| 34113 | 0.573 | 34423 | 0.399 | 35233 | 0.333 | 35543 | 0.181 |
| 34114 | 0.457 | 34424 | 0.283 | 35234 | 0.217 | 35544 | 0.065 |
| 34115 | 0.336 | 34425 | 0.163 | 35235 | 0.096 | 35545 | -0.055 |
| 34121 | 0.605 | 34431 | 0.483 | 35241 | 0.351 | 35551 | 0.158 |
| 34122 | 0.525 | 34432 | 0.403 | 35242 | 0.271 | 35552 | 0.078 |
| 34123 | 0.504 | 34433 | 0.382 | 35243 | 0.250 | 35553 | 0.057 |
| 34124 | 0.388 | 34434 | 0.266 | 35244 | 0.134 | 35554 | -0.059 |
| 34125 | 0.268 | 34435 | 0.146 | 35245 | 0.014 | 35555 | -0.179 |
| 34131 | 0.588 | 34441 | 0.400 | 35251 | 0.227 | 41111 | 0.852 |
| 34132 | 0.508 | 34442 | 0.320 | 35252 | 0.147 | 41112 | 0.772 |
| 34133 | 0.487 | 34443 | 0.300 | 35253 | 0.126 | 41113 | 0.751 |
| 34134 | 0.371 | 34444 | 0.184 | 35254 | 0.010 | 41114 | 0.635 |
| 34135 | 0.251 | 34445 | 0.063 | 35255 | -0.110 | 41115 | 0.515 |
| 34141 | 0.505 | 34451 | 0.276 | 35311 | 0.502 | 41121 | 0.783 |
| 34142 | 0.425 | 34452 | 0.196 | 35312 | 0.422 | 41122 | 0.703 |
| 34143 | 0.404 | 34453 | 0.176 | 35313 | 0.401 | 41123 | 0.682 |
| 34144 | 0.288 | 34454 | 0.060 | 35314 | 0.285 | 41124 | 0.566 |
| 34145 | 0.168 | 34455 | -0.061 | 35315 | 0.165 | 41125 | 0.446 |
| 34151 | 0.381 | 34511 | 0.534 | 35321 | 0.433 | 41131 | 0.766 |
| 34152 | 0.301 | 34512 | 0.454 | 35322 | 0.353 | 41132 | 0.686 |
| 34153 | 0.280 | 34513 | 0.433 | 35323 | 0.332 | 41133 | 0.665 |
| 34154 | 0.165 | 34514 | 0.317 | 35324 | 0.216 | 41134 | 0.549 |
| 34155 | 0.044 | 34515 | 0.197 | 35325 | 0.096 | 41135 | 0.429 |
| 34211 | 0.603 | 34521 | 0.465 | 35331 | 0.416 | 41141 | 0.683 |
| 34212 | 0.523 | 34522 | 0.385 | 35332 | 0.336 | 41142 | 0.603 |
| 34213 | 0.502 | 34523 | 0.364 | 35333 | 0.315 | 41143 | 0.583 |
| 34214 | 0.386 | 34524 | 0.248 | 35334 | 0.199 | 41144 | 0.467 |
| 34215 | 0.266 | 34525 | 0.128 | 35335 | 0.079 | 41145 | 0.346 |
| 34221 | 0.534 | 34531 | 0.448 | 35341 | 0.333 | 41151 | 0.559 |
| 34222 | 0.454 | 34532 | 0.368 | 35342 | 0.254 | 41152 | 0.480 |
| 34223 | 0.434 | 34533 | 0.347 | 35343 | 0.233 | 41153 | 0.459 |
| 34224 | 0.318 | 34534 | 0.231 | 35344 | 0.117 | 41154 | 0.343 |
| 34225 | 0.197 | 34535 | 0.111 | 35345 | -0.004 | 41155 | 0.222 |
| 34231 | 0.517 | 34541 | 0.366 | 35351 | 0.210 | 41211 | 0.781 |
| 34232 | 0.437 | 34542 | 0.286 | 35352 | 0.130 | 41212 | 0.701 |
| 34233 | 0.416 | 34543 | 0.265 | 35353 | 0.109 | 41213 | 0.681 |
| 34234 | 0.300 | 34544 | 0.149 | 35354 | -0.007 | 41214 | 0.565 |
| 34235 | 0.180 | 34545 | 0.029 | 35355 | -0.128 | 41215 | 0.444 |
| 34241 | 0.435 | 34551 | 0.242 | 35411 | 0.485 | 41221 | 0.712 |
| 34242 | 0.355 | 34552 | 0.162 | 35412 | 0.405 | 41222 | 0.633 |
| 34243 | 0.334 | 34553 | 0.141 | 35413 | 0.384 | 41223 | 0.612 |
| 34244 | 0.218 | 34554 | 0.025 | 35414 | 0.268 | 41224 | 0.496 |
| 34245 | 0.098 | 34555 | -0.095 | 35415 | 0.148 | 41225 | 0.375 |
| 34251 | 0.311 | 35111 | 0.590 | 35421 | 0.416 | 41231 | 0.695 |
| 34252 | 0.231 | 35112 | 0.510 | 35422 | 0.336 | 41232 | 0.616 |
| 34253 | 0.210 | 35113 | 0.489 | 35423 | 0.315 | 41233 | 0.595 |
| 34254 | 0.094 | 35114 | 0.373 | 35424 | 0.199 | 41234 | 0.479 |
| 34255 | -0.026 | 35115 | 0.253 | 35425 | 0.079 | 41235 | 0.358 |
| 34311 | 0.586 | 35121 | 0.521 | 35431 | 0.399 | 41241 | 0.613 |
| 34312 | 0.506 | 35122 | 0.441 | 35432 | 0.319 | 41242 | 0.533 |
| 34313 | 0.485 | 35123 | 0.420 | 35433 | 0.298 | 41243 | 0.512 |
| 34314 | 0.369 | 35124 | 0.304 | 35434 | 0.182 | 41244 | 0.396 |
| 34315 | 0.249 | 35125 | 0.184 | 35435 | 0.062 | 41245 | 0.276 |

| Health  state | Utility value | Health  state | Utility value | Health state | Utility value | Health  state | Utility value |
| --- | --- | --- | --- | --- | --- | --- | --- |
| 41251 | 0.489 | 42111 | 0.703 | 42421 | 0.529 | 43231 | 0.517 |
| 41252 | 0.409 | 42112 | 0.623 | 42422 | 0.449 | 43232 | 0.437 |
| 41253 | 0.388 | 42113 | 0.602 | 42423 | 0.429 | 43233 | 0.416 |
| 41254 | 0.272 | 42114 | 0.486 | 42424 | 0.313 | 43234 | 0.300 |
| 41255 | 0.152 | 42115 | 0.366 | 42425 | 0.192 | 43235 | 0.180 |
| 41311 | 0.764 | 42121 | 0.634 | 42431 | 0.512 | 43241 | 0.434 |
| 41312 | 0.684 | 42122 | 0.554 | 42432 | 0.432 | 43242 | 0.354 |
| 41313 | 0.663 | 42123 | 0.533 | 42433 | 0.411 | 43243 | 0.333 |
| 41314 | 0.547 | 42124 | 0.417 | 42434 | 0.295 | 43244 | 0.217 |
| 41315 | 0.427 | 42125 | 0.297 | 42435 | 0.175 | 43245 | 0.097 |
| 41321 | 0.695 | 42131 | 0.617 | 42441 | 0.430 | 43251 | 0.310 |
| 41322 | 0.615 | 42132 | 0.537 | 42442 | 0.350 | 43252 | 0.230 |
| 41323 | 0.594 | 42133 | 0.516 | 42443 | 0.329 | 43253 | 0.209 |
| 41324 | 0.478 | 42134 | 0.400 | 42444 | 0.213 | 43254 | 0.094 |
| 41325 | 0.358 | 42135 | 0.280 | 42445 | 0.093 | 43255 | -0.027 |
| 41331 | 0.678 | 42141 | 0.534 | 42451 | 0.306 | 43311 | 0.585 |
| 41332 | 0.598 | 42142 | 0.455 | 42452 | 0.226 | 43312 | 0.505 |
| 41333 | 0.577 | 42143 | 0.434 | 42453 | 0.205 | 43313 | 0.484 |
| 41334 | 0.461 | 42144 | 0.318 | 42454 | 0.089 | 43314 | 0.368 |
| 41335 | 0.341 | 42145 | 0.197 | 42455 | -0.031 | 43315 | 0.248 |
| 41341 | 0.596 | 42151 | 0.411 | 42511 | 0.563 | 43321 | 0.516 |
| 41342 | 0.516 | 42152 | 0.331 | 42512 | 0.483 | 43322 | 0.436 |
| 41343 | 0.495 | 42153 | 0.310 | 42513 | 0.463 | 43323 | 0.416 |
| 41344 | 0.379 | 42154 | 0.194 | 42514 | 0.347 | 43324 | 0.300 |
| 41345 | 0.259 | 42155 | 0.074 | 42515 | 0.226 | 43325 | 0.179 |
| 41351 | 0.472 | 42211 | 0.632 | 42521 | 0.494 | 43331 | 0.499 |
| 41352 | 0.392 | 42212 | 0.553 | 42522 | 0.415 | 43332 | 0.419 |
| 41353 | 0.371 | 42213 | 0.532 | 42523 | 0.394 | 43333 | 0.399 |
| 41354 | 0.255 | 42214 | 0.416 | 42524 | 0.278 | 43334 | 0.283 |
| 41355 | 0.135 | 42215 | 0.295 | 42525 | 0.157 | 43335 | 0.162 |
| 41411 | 0.747 | 42221 | 0.564 | 42531 | 0.477 | 43341 | 0.417 |
| 41412 | 0.667 | 42222 | 0.484 | 42532 | 0.397 | 43342 | 0.337 |
| 41413 | 0.646 | 42223 | 0.463 | 42533 | 0.377 | 43343 | 0.316 |
| 41414 | 0.530 | 42224 | 0.347 | 42534 | 0.261 | 43344 | 0.200 |
| 41415 | 0.410 | 42225 | 0.227 | 42535 | 0.140 | 43345 | 0.080 |
| 41421 | 0.678 | 42231 | 0.547 | 42541 | 0.395 | 43351 | 0.293 |
| 41422 | 0.598 | 42232 | 0.467 | 42542 | 0.315 | 43352 | 0.213 |
| 41423 | 0.577 | 42233 | 0.446 | 42543 | 0.294 | 43353 | 0.192 |
| 41424 | 0.461 | 42234 | 0.330 | 42544 | 0.178 | 43354 | 0.076 |
| 41425 | 0.341 | 42235 | 0.210 | 42545 | 0.058 | 43355 | -0.044 |
| 41431 | 0.661 | 42241 | 0.464 | 42551 | 0.271 | 43411 | 0.568 |
| 41432 | 0.581 | 42242 | 0.384 | 42552 | 0.191 | 43412 | 0.488 |
| 41433 | 0.560 | 42243 | 0.363 | 42553 | 0.170 | 43413 | 0.467 |
| 41434 | 0.444 | 42244 | 0.247 | 42554 | 0.054 | 43414 | 0.351 |
| 41435 | 0.324 | 42245 | 0.127 | 42555 | -0.066 | 43415 | 0.231 |
| 41441 | 0.579 | 42251 | 0.340 | 43111 | 0.673 | 43421 | 0.499 |
| 41442 | 0.499 | 42252 | 0.260 | 43112 | 0.593 | 43422 | 0.419 |
| 41443 | 0.478 | 42253 | 0.239 | 43113 | 0.572 | 43423 | 0.399 |
| 41444 | 0.362 | 42254 | 0.123 | 43114 | 0.456 | 43424 | 0.283 |
| 41445 | 0.242 | 42255 | 0.003 | 43115 | 0.336 | 43425 | 0.162 |
| 41451 | 0.455 | 42311 | 0.615 | 43121 | 0.604 | 43431 | 0.482 |
| 41452 | 0.375 | 42312 | 0.535 | 43122 | 0.524 | 43432 | 0.402 |
| 41453 | 0.354 | 42313 | 0.514 | 43123 | 0.503 | 43433 | 0.382 |
| 41454 | 0.238 | 42314 | 0.398 | 43124 | 0.387 | 43434 | 0.266 |
| 41455 | 0.118 | 42315 | 0.278 | 43125 | 0.267 | 43435 | 0.145 |
| 41511 | 0.712 | 42321 | 0.546 | 43131 | 0.587 | 43441 | 0.400 |
| 41512 | 0.632 | 42322 | 0.466 | 43132 | 0.507 | 43442 | 0.320 |
| 41513 | 0.611 | 42323 | 0.446 | 43133 | 0.486 | 43443 | 0.299 |
| 41514 | 0.495 | 42324 | 0.330 | 43134 | 0.370 | 43444 | 0.183 |
| 41515 | 0.375 | 42325 | 0.209 | 43135 | 0.250 | 43445 | 0.063 |
| 41521 | 0.643 | 42331 | 0.529 | 43141 | 0.505 | 43451 | 0.276 |
| 41522 | 0.563 | 42332 | 0.449 | 43142 | 0.425 | 43452 | 0.196 |
| 41523 | 0.543 | 42333 | 0.428 | 43143 | 0.404 | 43453 | 0.175 |
| 41524 | 0.427 | 42334 | 0.313 | 43144 | 0.288 | 43454 | 0.059 |
| 41525 | 0.306 | 42335 | 0.192 | 43145 | 0.168 | 43455 | -0.061 |
| 41531 | 0.626 | 42341 | 0.447 | 43151 | 0.381 | 43511 | 0.533 |
| 41532 | 0.546 | 42342 | 0.367 | 43152 | 0.301 | 43512 | 0.453 |
| 41533 | 0.526 | 42343 | 0.346 | 43153 | 0.280 | 43513 | 0.433 |
| 41534 | 0.410 | 42344 | 0.230 | 43154 | 0.164 | 43514 | 0.317 |
| 41535 | 0.289 | 42345 | 0.110 | 43155 | 0.044 | 43515 | 0.196 |
| 41541 | 0.544 | 42351 | 0.323 | 43211 | 0.602 | 43521 | 0.465 |
| 41542 | 0.464 | 42352 | 0.243 | 43212 | 0.523 | 43522 | 0.385 |
| 41543 | 0.443 | 42353 | 0.222 | 43213 | 0.502 | 43523 | 0.364 |
| 41544 | 0.327 | 42354 | 0.106 | 43214 | 0.386 | 43524 | 0.248 |
| 41545 | 0.207 | 42355 | -0.014 | 43215 | 0.265 | 43525 | 0.128 |
| 41551 | 0.420 | 42411 | 0.598 | 43221 | 0.534 | 43531 | 0.447 |
| 41552 | 0.340 | 42412 | 0.518 | 43222 | 0.454 | 43532 | 0.368 |
| 41553 | 0.319 | 42413 | 0.497 | 43223 | 0.433 | 43533 | 0.347 |
| 41554 | 0.203 | 42414 | 0.381 | 43224 | 0.317 | 43534 | 0.231 |
| 41555 | 0.083 | 42415 | 0.261 | 43225 | 0.197 | 43535 | 0.110 |

| Health  state | Utility value | Health  state | Utility value | Health state | Utility value | Health  state | Utility value |
| --- | --- | --- | --- | --- | --- | --- | --- |
| 43541 | 0.365 | 44351 | 0.266 | 45211 | 0.492 | 45521 | 0.354 |
| 43542 | 0.285 | 44352 | 0.186 | 45212 | 0.412 | 45522 | 0.274 |
| 43543 | 0.264 | 44353 | 0.165 | 45213 | 0.391 | 45523 | 0.253 |
| 43544 | 0.148 | 44354 | 0.049 | 45214 | 0.275 | 45524 | 0.137 |
| 43545 | 0.028 | 44355 | -0.071 | 45215 | 0.155 | 45525 | 0.017 |
| 43551 | 0.241 | 44411 | 0.541 | 45221 | 0.423 | 45531 | 0.337 |
| 43552 | 0.161 | 44412 | 0.461 | 45222 | 0.343 | 45532 | 0.257 |
| 43553 | 0.140 | 44413 | 0.440 | 45223 | 0.322 | 45533 | 0.236 |
| 43554 | 0.024 | 44414 | 0.325 | 45224 | 0.206 | 45534 | 0.120 |
| 43555 | -0.096 | 44415 | 0.204 | 45225 | 0.086 | 45535 | 0.000 |
| 44111 | 0.646 | 44421 | 0.472 | 45231 | 0.406 | 45541 | 0.254 |
| 44112 | 0.566 | 44422 | 0.392 | 45232 | 0.326 | 45542 | 0.174 |
| 44113 | 0.545 | 44423 | 0.372 | 45233 | 0.305 | 45543 | 0.153 |
| 44114 | 0.429 | 44424 | 0.256 | 45234 | 0.189 | 45544 | 0.038 |
| 44115 | 0.309 | 44425 | 0.135 | 45235 | 0.069 | 45545 | -0.083 |
| 44121 | 0.577 | 44431 | 0.455 | 45241 | 0.323 | 45551 | 0.130 |
| 44122 | 0.497 | 44432 | 0.375 | 45242 | 0.243 | 45552 | 0.050 |
| 44123 | 0.476 | 44433 | 0.355 | 45243 | 0.223 | 45553 | 0.030 |
| 44124 | 0.361 | 44434 | 0.239 | 45244 | 0.107 | 45554 | -0.086 |
| 44125 | 0.240 | 44435 | 0.118 | 45245 | -0.014 | 45555 | -0.207 |
| 44131 | 0.560 | 44441 | 0.373 | 45251 | 0.199 | 51111 | 0.498 |
| 44132 | 0.480 | 44442 | 0.293 | 45252 | 0.120 | 51112 | 0.418 |
| 44133 | 0.459 | 44443 | 0.272 | 45253 | 0.099 | 51113 | 0.398 |
| 44134 | 0.343 | 44444 | 0.156 | 45254 | -0.017 | 51114 | 0.282 |
| 44135 | 0.223 | 44445 | 0.036 | 45255 | -0.138 | 51115 | 0.161 |
| 44141 | 0.478 | 44451 | 0.249 | 45311 | 0.474 | 51121 | 0.429 |
| 44142 | 0.398 | 44452 | 0.169 | 45312 | 0.394 | 51122 | 0.350 |
| 44143 | 0.377 | 44453 | 0.148 | 45313 | 0.374 | 51123 | 0.329 |
| 44144 | 0.261 | 44454 | 0.032 | 45314 | 0.258 | 51124 | 0.213 |
| 44145 | 0.141 | 44455 | -0.088 | 45315 | 0.137 | 51125 | 0.092 |
| 44151 | 0.354 | 44511 | 0.506 | 45321 | 0.406 | 51131 | 0.412 |
| 44152 | 0.274 | 44512 | 0.427 | 45322 | 0.326 | 51132 | 0.333 |
| 44153 | 0.253 | 44513 | 0.406 | 45323 | 0.305 | 51133 | 0.312 |
| 44154 | 0.137 | 44514 | 0.290 | 45324 | 0.189 | 51134 | 0.196 |
| 44155 | 0.017 | 44515 | 0.169 | 45325 | 0.069 | 51135 | 0.075 |
| 44211 | 0.576 | 44521 | 0.438 | 45331 | 0.388 | 51141 | 0.330 |
| 44212 | 0.496 | 44522 | 0.358 | 45332 | 0.309 | 51142 | 0.250 |
| 44213 | 0.475 | 44523 | 0.337 | 45333 | 0.288 | 51143 | 0.229 |
| 44214 | 0.359 | 44524 | 0.221 | 45334 | 0.172 | 51144 | 0.113 |
| 44215 | 0.239 | 44525 | 0.101 | 45335 | 0.051 | 51145 | -0.007 |
| 44221 | 0.507 | 44531 | 0.421 | 45341 | 0.306 | 51151 | 0.206 |
| 44222 | 0.427 | 44532 | 0.341 | 45342 | 0.226 | 51152 | 0.126 |
| 44223 | 0.406 | 44533 | 0.320 | 45343 | 0.205 | 51153 | 0.105 |
| 44224 | 0.290 | 44534 | 0.204 | 45344 | 0.089 | 51154 | -0.011 |
| 44225 | 0.170 | 44535 | 0.084 | 45345 | -0.031 | 51155 | -0.131 |
| 44231 | 0.490 | 44541 | 0.338 | 45351 | 0.182 | 51211 | 0.428 |
| 44232 | 0.410 | 44542 | 0.258 | 45352 | 0.102 | 51212 | 0.348 |
| 44233 | 0.389 | 44543 | 0.237 | 45353 | 0.081 | 51213 | 0.327 |
| 44234 | 0.273 | 44544 | 0.121 | 45354 | -0.035 | 51214 | 0.211 |
| 44235 | 0.153 | 44545 | 0.001 | 45355 | -0.155 | 51215 | 0.091 |
| 44241 | 0.407 | 44551 | 0.214 | 45411 | 0.457 | 51221 | 0.359 |
| 44242 | 0.327 | 44552 | 0.134 | 45412 | 0.377 | 51222 | 0.279 |
| 44243 | 0.307 | 44553 | 0.113 | 45413 | 0.357 | 51223 | 0.258 |
| 44244 | 0.191 | 44554 | -0.003 | 45414 | 0.241 | 51224 | 0.142 |
| 44245 | 0.070 | 44555 | -0.123 | 45415 | 0.120 | 51225 | 0.022 |
| 44251 | 0.283 | 45111 | 0.562 | 45421 | 0.389 | 51231 | 0.342 |
| 44252 | 0.203 | 45112 | 0.482 | 45422 | 0.309 | 51232 | 0.262 |
| 44253 | 0.183 | 45113 | 0.461 | 45423 | 0.288 | 51233 | 0.241 |
| 44254 | 0.067 | 45114 | 0.345 | 45424 | 0.172 | 51234 | 0.125 |
| 44255 | -0.054 | 45115 | 0.225 | 45425 | 0.052 | 51235 | 0.005 |
| 44311 | 0.558 | 45121 | 0.493 | 45431 | 0.371 | 51241 | 0.260 |
| 44312 | 0.478 | 45122 | 0.413 | 45432 | 0.292 | 51242 | 0.180 |
| 44313 | 0.458 | 45123 | 0.393 | 45433 | 0.271 | 51243 | 0.159 |
| 44314 | 0.342 | 45124 | 0.277 | 45434 | 0.155 | 51244 | 0.043 |
| 44315 | 0.221 | 45125 | 0.156 | 45435 | 0.034 | 51245 | -0.077 |
| 44321 | 0.489 | 45131 | 0.476 | 45441 | 0.289 | 51251 | 0.136 |
| 44322 | 0.409 | 45132 | 0.396 | 45442 | 0.209 | 51252 | 0.056 |
| 44323 | 0.389 | 45133 | 0.376 | 45443 | 0.188 | 51253 | 0.035 |
| 44324 | 0.273 | 45134 | 0.260 | 45444 | 0.072 | 51254 | -0.081 |
| 44325 | 0.152 | 45135 | 0.139 | 45445 | -0.048 | 51255 | -0.201 |
| 44331 | 0.472 | 45141 | 0.394 | 45451 | 0.165 | 51311 | 0.411 |
| 44332 | 0.392 | 45142 | 0.314 | 45452 | 0.085 | 51312 | 0.331 |
| 44333 | 0.372 | 45143 | 0.293 | 45453 | 0.064 | 51313 | 0.310 |
| 44334 | 0.256 | 45144 | 0.177 | 45454 | -0.052 | 51314 | 0.194 |
| 44335 | 0.135 | 45145 | 0.057 | 45455 | -0.172 | 51315 | 0.074 |
| 44341 | 0.390 | 45151 | 0.270 | 45511 | 0.423 | 51321 | 0.342 |
| 44342 | 0.310 | 45152 | 0.190 | 45512 | 0.343 | 51322 | 0.262 |
| 44343 | 0.289 | 45153 | 0.169 | 45513 | 0.322 | 51323 | 0.241 |
| 44344 | 0.173 | 45154 | 0.053 | 45514 | 0.206 | 51324 | 0.125 |
| 44345 | 0.053 | 45155 | -0.067 | 45515 | 0.086 | 51325 | 0.005 |

| Health  state | Utility value | Health  state | Utility value | Health state | Utility value | Health  state | Utility value |
| --- | --- | --- | --- | --- | --- | --- | --- |
| 51331 | 0.325 | 52141 | 0.181 | 52451 | -0.048 | 53311 | 0.232 |
| 51332 | 0.245 | 52142 | 0.101 | 52452 | -0.128 | 53312 | 0.152 |
| 51333 | 0.224 | 52143 | 0.080 | 52453 | -0.148 | 53313 | 0.131 |
| 51334 | 0.108 | 52144 | -0.036 | 52454 | -0.264 | 53314 | 0.015 |
| 51335 | -0.012 | 52145 | -0.156 | 52455 | -0.385 | 53315 | -0.105 |
| 51341 | 0.242 | 52151 | 0.057 | 52511 | 0.210 | 53321 | 0.163 |
| 51342 | 0.162 | 52152 | -0.023 | 52512 | 0.130 | 53322 | 0.083 |
| 51343 | 0.141 | 52153 | -0.044 | 52513 | 0.109 | 53323 | 0.062 |
| 51344 | 0.025 | 52154 | -0.160 | 52514 | -0.007 | 53324 | -0.054 |
| 51345 | -0.095 | 52155 | -0.280 | 52515 | -0.127 | 53325 | -0.174 |
| 51351 | 0.118 | 52211 | 0.279 | 52521 | 0.141 | 53331 | 0.146 |
| 51352 | 0.038 | 52212 | 0.199 | 52522 | 0.061 | 53332 | 0.066 |
| 51353 | 0.018 | 52213 | 0.178 | 52523 | 0.040 | 53333 | 0.045 |
| 51354 | -0.098 | 52214 | 0.062 | 52524 | -0.076 | 53334 | -0.071 |
| 51355 | -0.219 | 52215 | -0.058 | 52525 | -0.196 | 53335 | -0.191 |
| 51411 | 0.393 | 52221 | 0.210 | 52531 | 0.124 | 53341 | 0.063 |
| 51412 | 0.314 | 52222 | 0.130 | 52532 | 0.044 | 53342 | -0.017 |
| 51413 | 0.293 | 52223 | 0.109 | 52533 | 0.023 | 53343 | -0.037 |
| 51414 | 0.177 | 52224 | -0.006 | 52534 | -0.093 | 53344 | -0.153 |
| 51415 | 0.056 | 52225 | -0.127 | 52535 | -0.213 | 53345 | -0.274 |
| 51421 | 0.325 | 52231 | 0.193 | 52541 | 0.041 | 53351 | -0.061 |
| 51422 | 0.245 | 52232 | 0.113 | 52542 | -0.038 | 53352 | -0.140 |
| 51423 | 0.224 | 52233 | 0.092 | 52543 | -0.059 | 53353 | -0.161 |
| 51424 | 0.108 | 52234 | -0.024 | 52544 | -0.175 | 53354 | -0.277 |
| 51425 | -0.012 | 52235 | -0.144 | 52545 | -0.296 | 53355 | -0.398 |
| 51431 | 0.308 | 52241 | 0.111 | 52551 | -0.082 | 53411 | 0.215 |
| 51432 | 0.228 | 52242 | 0.031 | 52552 | -0.162 | 53412 | 0.135 |
| 51433 | 0.207 | 52243 | 0.010 | 52553 | -0.183 | 53413 | 0.114 |
| 51434 | 0.091 | 52244 | -0.106 | 52554 | -0.299 | 53414 | -0.002 |
| 51435 | -0.029 | 52245 | -0.226 | 52555 | -0.419 | 53415 | -0.122 |
| 51441 | 0.225 | 52251 | -0.013 | 53111 | 0.320 | 53421 | 0.146 |
| 51442 | 0.145 | 52252 | -0.093 | 53112 | 0.240 | 53422 | 0.066 |
| 51443 | 0.124 | 52253 | -0.114 | 53113 | 0.219 | 53423 | 0.045 |
| 51444 | 0.008 | 52254 | -0.230 | 53114 | 0.103 | 53424 | -0.071 |
| 51445 | -0.112 | 52255 | -0.350 | 53115 | -0.018 | 53425 | -0.191 |
| 51451 | 0.101 | 52311 | 0.262 | 53121 | 0.251 | 53431 | 0.129 |
| 51452 | 0.021 | 52312 | 0.182 | 53122 | 0.171 | 53432 | 0.049 |
| 51453 | 0.000 | 52313 | 0.161 | 53123 | 0.150 | 53433 | 0.028 |
| 51454 | -0.115 | 52314 | 0.045 | 53124 | 0.034 | 53434 | -0.088 |
| 51455 | -0.236 | 52315 | -0.075 | 53125 | -0.086 | 53435 | -0.208 |
| 51511 | 0.359 | 52321 | 0.193 | 53131 | 0.234 | 53441 | 0.046 |
| 51512 | 0.279 | 52322 | 0.113 | 53132 | 0.154 | 53442 | -0.034 |
| 51513 | 0.258 | 52323 | 0.092 | 53133 | 0.133 | 53443 | -0.054 |
| 51514 | 0.142 | 52324 | -0.024 | 53134 | 0.017 | 53444 | -0.170 |
| 51515 | 0.022 | 52325 | -0.144 | 53135 | -0.103 | 53445 | -0.291 |
| 51521 | 0.290 | 52331 | 0.176 | 53141 | 0.151 | 53451 | -0.078 |
| 51522 | 0.210 | 52332 | 0.096 | 53142 | 0.071 | 53452 | -0.158 |
| 51523 | 0.189 | 52333 | 0.075 | 53143 | 0.050 | 53453 | -0.178 |
| 51524 | 0.073 | 52334 | -0.041 | 53144 | -0.066 | 53454 | -0.294 |
| 51525 | -0.047 | 52335 | -0.161 | 53145 | -0.186 | 53455 | -0.415 |
| 51531 | 0.273 | 52341 | 0.093 | 53151 | 0.027 | 53511 | 0.180 |
| 51532 | 0.193 | 52342 | 0.013 | 53152 | -0.053 | 53512 | 0.100 |
| 51533 | 0.172 | 52343 | -0.007 | 53153 | -0.074 | 53513 | 0.079 |
| 51534 | 0.056 | 52344 | -0.123 | 53154 | -0.189 | 53514 | -0.037 |
| 51535 | -0.064 | 52345 | -0.244 | 53155 | -0.310 | 53515 | -0.157 |
| 51541 | 0.190 | 52351 | -0.031 | 53211 | 0.249 | 53521 | 0.111 |
| 51542 | 0.110 | 52352 | -0.111 | 53212 | 0.169 | 53522 | 0.031 |
| 51543 | 0.090 | 52353 | -0.131 | 53213 | 0.148 | 53523 | 0.010 |
| 51544 | -0.026 | 52354 | -0.247 | 53214 | 0.032 | 53524 | -0.106 |
| 51545 | -0.147 | 52355 | -0.368 | 53215 | -0.088 | 53525 | -0.226 |
| 51551 | 0.066 | 52411 | 0.245 | 53221 | 0.180 | 53531 | 0.094 |
| 51552 | -0.013 | 52412 | 0.165 | 53222 | 0.100 | 53532 | 0.014 |
| 51553 | -0.034 | 52413 | 0.144 | 53223 | 0.080 | 53533 | -0.007 |
| 51554 | -0.150 | 52414 | 0.028 | 53224 | -0.036 | 53534 | -0.123 |
| 51555 | -0.271 | 52415 | -0.092 | 53225 | -0.157 | 53535 | -0.243 |
| 52111 | 0.349 | 52421 | 0.176 | 53231 | 0.163 | 53541 | 0.012 |
| 52112 | 0.270 | 52422 | 0.096 | 53232 | 0.083 | 53542 | -0.068 |
| 52113 | 0.249 | 52423 | 0.075 | 53233 | 0.062 | 53543 | -0.089 |
| 52114 | 0.133 | 52424 | -0.041 | 53234 | -0.053 | 53544 | -0.205 |
| 52115 | 0.012 | 52425 | -0.161 | 53235 | -0.174 | 53545 | -0.325 |
| 52121 | 0.281 | 52431 | 0.159 | 53241 | 0.081 | 53551 | -0.112 |
| 52122 | 0.201 | 52432 | 0.079 | 53242 | 0.001 | 53552 | -0.192 |
| 52123 | 0.180 | 52433 | 0.058 | 53243 | -0.020 | 53553 | -0.213 |
| 52124 | 0.064 | 52434 | -0.058 | 53244 | -0.136 | 53554 | -0.329 |
| 52125 | -0.056 | 52435 | -0.178 | 53245 | -0.256 | 53555 | -0.449 |
| 52131 | 0.264 | 52441 | 0.076 | 53251 | -0.043 | 54111 | 0.293 |
| 52132 | 0.184 | 52442 | -0.004 | 53252 | -0.123 | 54112 | 0.213 |
| 52133 | 0.163 | 52443 | -0.024 | 53253 | -0.144 | 54113 | 0.192 |
| 52134 | 0.047 | 52444 | -0.140 | 53254 | -0.260 | 54114 | 0.076 |
| 52135 | -0.073 | 52445 | -0.261 | 53255 | -0.380 | 54115 | -0.044 |

| Health  state | Utility value | Health  state | Utility value | Health state | Utility value | Health  state | Utility value |
| --- | --- | --- | --- | --- | --- | --- | --- |
| 54121 | 0.224 | 54431 | 0.102 | 55241 | -0.030 | 55551 | -0.223 |
| 54122 | 0.144 | 54432 | 0.022 | 55242 | -0.110 | 55552 | -0.303 |
| 54123 | 0.123 | 54433 | 0.001 | 55243 | -0.131 | 55553 | -0.324 |
| 54124 | 0.007 | 54434 | -0.115 | 55244 | -0.247 | 55554 | -0.440 |
| 54125 | -0.113 | 54435 | -0.235 | 55245 | -0.367 | 55555 | -0.560 |
| 54131 | 0.207 | 54441 | 0.019 | 55251 | -0.154 |  |  |
| 54132 | 0.127 | 54442 | -0.060 | 55252 | -0.234 |  |  |
| 54133 | 0.106 | 54443 | -0.081 | 55253 | -0.255 |  |  |
| 54134 | -0.010 | 54444 | -0.197 | 55254 | -0.371 |  |  |
| 54135 | -0.130 | 54445 | -0.318 | 55255 | -0.491 |  |  |
| 54141 | 0.124 | 54451 | -0.105 | 55311 | 0.121 |  |  |
| 54142 | 0.044 | 54452 | -0.184 | 55312 | 0.041 |  |  |
| 54143 | 0.024 | 54453 | -0.205 | 55313 | 0.020 |  |  |
| 54144 | -0.092 | 54454 | -0.321 | 55314 | -0.096 |  |  |
| 54145 | -0.213 | 54455 | -0.442 | 55315 | -0.216 |  |  |
| 54151 | 0.000 | 54511 | 0.153 | 55321 | 0.052 |  |  |
| 54152 | -0.080 | 54512 | 0.073 | 55322 | -0.028 |  |  |
| 54153 | -0.100 | 54513 | 0.052 | 55323 | -0.049 |  |  |
| 54154 | -0.216 | 54514 | -0.064 | 55324 | -0.165 |  |  |
| 54155 | -0.337 | 54515 | -0.184 | 55325 | -0.285 |  |  |
| 54211 | 0.222 | 54521 | 0.084 | 55331 | 0.035 |  |  |
| 54212 | 0.142 | 54522 | 0.004 | 55332 | -0.045 |  |  |
| 54213 | 0.121 | 54523 | -0.017 | 55333 | -0.066 |  |  |
| 54214 | 0.005 | 54524 | -0.132 | 55334 | -0.182 |  |  |
| 54215 | -0.115 | 54525 | -0.253 | 55335 | -0.302 |  |  |
| 54221 | 0.153 | 54531 | 0.067 | 55341 | -0.047 |  |  |
| 54222 | 0.073 | 54532 | -0.013 | 55342 | -0.127 |  |  |
| 54223 | 0.053 | 54533 | -0.034 | 55343 | -0.148 |  |  |
| 54224 | -0.063 | 54534 | -0.150 | 55344 | -0.264 |  |  |
| 54225 | -0.184 | 54535 | -0.270 | 55345 | -0.384 |  |  |
| 54231 | 0.136 | 54541 | -0.015 | 55351 | -0.171 |  |  |
| 54232 | 0.056 | 54542 | -0.095 | 55352 | -0.251 |  |  |
| 54233 | 0.036 | 54543 | -0.116 | 55353 | -0.272 |  |  |
| 54234 | -0.080 | 54544 | -0.232 | 55354 | -0.388 |  |  |
| 54235 | -0.201 | 54545 | -0.352 | 55355 | -0.508 |  |  |
| 54241 | 0.054 | 54551 | -0.139 | 55411 | 0.104 |  |  |
| 54242 | -0.026 | 54552 | -0.219 | 55412 | 0.024 |  |  |
| 54243 | -0.047 | 54553 | -0.240 | 55413 | 0.003 |  |  |
| 54244 | -0.163 | 54554 | -0.356 | 55414 | -0.113 |  |  |
| 54245 | -0.283 | 54555 | -0.476 | 55415 | -0.233 |  |  |
| 54251 | -0.070 | 55111 | 0.209 | 55421 | 0.035 |  |  |
| 54252 | -0.150 | 55112 | 0.129 | 55422 | -0.045 |  |  |
| 54253 | -0.171 | 55113 | 0.108 | 55423 | -0.066 |  |  |
| 54254 | -0.287 | 55114 | -0.008 | 55424 | -0.182 |  |  |
| 54255 | -0.407 | 55115 | -0.128 | 55425 | -0.302 |  |  |
| 54311 | 0.205 | 55121 | 0.140 | 55431 | 0.018 |  |  |
| 54312 | 0.125 | 55122 | 0.060 | 55432 | -0.062 |  |  |
| 54313 | 0.104 | 55123 | 0.039 | 55433 | -0.083 |  |  |
| 54314 | -0.012 | 55124 | -0.077 | 55434 | -0.199 |  |  |
| 54315 | -0.132 | 55125 | -0.197 | 55435 | -0.319 |  |  |
| 54321 | 0.136 | 55131 | 0.123 | 55441 | -0.064 |  |  |
| 54322 | 0.056 | 55132 | 0.043 | 55442 | -0.144 |  |  |
| 54323 | 0.035 | 55133 | 0.022 | 55443 | -0.165 |  |  |
| 54324 | -0.081 | 55134 | -0.094 | 55444 | -0.281 |  |  |
| 54325 | -0.201 | 55135 | -0.214 | 55445 | -0.401 |  |  |
| 54331 | 0.119 | 55141 | 0.040 | 55451 | -0.188 |  |  |
| 54332 | 0.039 | 55142 | -0.040 | 55452 | -0.268 |  |  |
| 54333 | 0.018 | 55143 | -0.060 | 55453 | -0.289 |  |  |
| 54334 | -0.098 | 55144 | -0.176 | 55454 | -0.405 |  |  |
| 54335 | -0.218 | 55145 | -0.297 | 55455 | -0.525 |  |  |
| 54341 | 0.036 | 55151 | -0.084 | 55511 | 0.069 |  |  |
| 54342 | -0.043 | 55152 | -0.163 | 55512 | -0.011 |  |  |
| 54343 | -0.064 | 55153 | -0.184 | 55513 | -0.032 |  |  |
| 54344 | -0.180 | 55154 | -0.300 | 55514 | -0.148 |  |  |
| 54345 | -0.301 | 55155 | -0.421 | 55515 | -0.268 |  |  |
| 54351 | -0.088 | 55211 | 0.138 | 55521 | 0.000 |  |  |
| 54352 | -0.167 | 55212 | 0.058 | 55522 | -0.080 |  |  |
| 54353 | -0.188 | 55213 | 0.038 | 55523 | -0.100 |  |  |
| 54354 | -0.304 | 55214 | -0.078 | 55524 | -0.216 |  |  |
| 54355 | -0.425 | 55215 | -0.199 | 55525 | -0.337 |  |  |
| 54411 | 0.188 | 55221 | 0.069 | 55531 | -0.017 |  |  |
| 54412 | 0.108 | 55222 | -0.010 | 55532 | -0.097 |  |  |
| 54413 | 0.087 | 55223 | -0.031 | 55533 | -0.118 |  |  |
| 54414 | -0.029 | 55224 | -0.147 | 55534 | -0.233 |  |  |
| 54415 | -0.149 | 55225 | -0.268 | 55535 | -0.354 |  |  |
| 54421 | 0.119 | 55231 | 0.052 | 55541 | -0.099 |  |  |
| 54422 | 0.039 | 55232 | -0.028 | 55542 | -0.179 |  |  |
| 54423 | 0.018 | 55233 | -0.048 | 55543 | -0.200 |  |  |
| 54424 | -0.098 | 55234 | -0.164 | 55544 | -0.316 |  |  |
| 54425 | -0.218 | 55235 | -0.285 | 55545 | -0.436 |  |  |
